# Synthesis and Acaricidal Activities of Scopoletin Phenolic Ether Derivatives: QSAR, Molecular Docking Study and in Silico ADME Predictions

**DOI:** 10.3390/molecules23050995

**Published:** 2018-04-24

**Authors:** Jinxiang Luo, Ting Lai, Tao Guo, Fei Chen, Linli Zhang, Wei Ding, Yongqiang Zhang

**Affiliations:** College of Plant Protection, Southwest University, Chongqing 400715, China; xiangxiangnx@163.com (J.L.); laiting93@163.com (T. L.); 13994888326.guotao@163.com (T.G.); cf759974605@126.com (F.C.); zhll87_9@163.com (L. Z.); dwing818@163.com (W.D.)

**Keywords:** scopoletin, acaricidal activity, QSAR, molecular docking, ADME properties

## Abstract

Thirty phenolic ether derivatives of scopoletin modified at the 7-hydroxy position were synthesized, and their structures were confirmed by IR, ^1^H-NMR, ^13^C-NMR, MS and elemental analysis. Preliminary acaricidal activities of these compounds against female adults of *Tetranychus cinnabarinus* (Boisduval) were evaluated using the slide-dip method. The results indicated that some of these compounds exhibit more pronounced acaricidal activity than scopoletin, especially compounds **32**, **20**, **28**, **27** and **8** which exhibited about 8.41-, 7.32-, 7.23-, 6.76-, and 6.65-fold higher acaricidal potency. Compound **32** possessed the the most promising acaricidal activity and exhibited about 1.45-fold higher acaricidal potency against *T. cinnabarinus* than propargite. Statistically significant 2D-QSAR model supports the observed acaricidal activities and reveals that polarizability (HATS5p) was the most important parameter controlling bioactivity. 3D-QSAR (CoMFA: q^2^ = 0.802, r^2^ = 0.993; CoMSIA: q^2^ = 0.735, r^2^ = 0.965) results show that bulky substituents at R_4_, R_1_, R_2_ and R_5_ (C_6_, C_3_, C_4_, and C_7_) positions, electron positive groups at R_5_ (C_7_) position, hydrophobic groups at R_1_ (C_3_) and R_2_ (C_4_), H-bond donors groups at R_1_ (C_3_) and R_4_ (C_6_) will increase their acaricidal activity, which provide a good insight into the molecular features relevant to the acaricidal activity for further designing novel acaricidal agents. Molecular docking demonstrates that these selected derivatives display different bide modes with *TcPMCA1* from lead compound and they interact with more key amino acid residues than scopoletin. In silico ADME properties of scopoletin and its phenolic ether derivatives were also analyzed and showed potential to develop as good acaricidal candidates.

## 1. Introduction

The carmine spider mite, *Tetranychus cinnabarinus* (Boisduval), is considered as one of the most economically important arthropod pests [1]. This mite has been reported to infest over 100 crops or plants grown in the field or greenhouse worldwide, especially cotton, beans, eggplants, tomatoes, peppers, cucurbits and strawberries and so on [2,3,4]. Spider mites usually feed through a piercing-sucking process to remove cellular contents, resulting in reduction of photosynthesis and transpiration rates in plants [5,6]. The plants slightly infested by spider mite display discoloration of their leaves and defoliation, bud and fruit dropping and reductions in fruit yield and quality; serious plants infestations by this mite will cause whole plant death [7]. It is recognized as one of the most difficult mites to control mainly due to its small size, high reproductive potential, extremely short life cycle, and strong adaptability and ability to develop resistance [8,9]. The genetic system of spider mites is known as arrhenotoky, wherein unfertilized haploid eggs develop into males and fertilized eggs develop into females [6]. This type of genetic system is highly vulnerable to mutations, conferring resistance to acaricides [10]. In addition, for a long time, this pest mite was controlled mainly through frequent applications of synthetic chemical acaricides, which have resulted in mite resistance to almost all major classes of acaricides throughout the world as well as environmental problems [11,12,13,14,15]. Therefore, it is necessary to develop novel, powerful, and environmentally-friendly acaricides from natural products, which will be used as an alternative agent to control this pest mite.

Scopoletin is an important secondary metabolite found in many plant species, such as *Erycibe obtusifolia* Benth [16], *Aster tataricus* [17], *Foeniculum vulgare* [18], *Artemisia annua* L. [19], *Sinomonium acutum* [20], and *Melia azedarach* L. fruits [21]. Scopoletin is classified as a coumarin and chemically known as 7-hydroxy-6-methoxy-2*H*-chromen-2-one [22]. Studies have shown that scopoletin has a wide spectrum of biological activities, such as pronounced acaricidal [23,24], anti-inflammatory [25,26], antitumoral [27], antioxidative [20], hepatoprotective [28], insecticidal [29], antifungal [30], and alleopathic properties [31]. Based on its pronounced acaricidal activities, our research group further investigated the mechanism of action and found that Ca^2+^-ATPase, which is vital in nervous signal conduction [32,33,34], was inhibited [35] and *TcPMCA1* from Ca^2+^-ATPase was significantly upregulated after *T. cinnabarlnus* was exposed to scopoletin, and molecular docking also showed that scopoletin inserts into the binding cavity and interacts with *TcPMCA1* protein through the driving forces of hydrogen bonds [36]. However, its acaricidal activity remain lower than that of some registered synthetic chemical acaricides, such as pyridaben. To date, few studies have attempted to improve the acaricidal effects of scopoletin by modifying its structure.

Quantitative structure-activity relationship (QSAR) and molecular docking are two important computational approaches, which have been considered as effective facilitating tools in drug design and discovery [37,38]. Quantitative structure-activity relationship (QSAR) is a method that correlates chemical structure of the compound with its biological activity [39,40,41]. QSAR has also been widely used to provide useful information for the design and discovery of insecticidal and acaricidal agents [42,43]. Molecular docking is a computational method to identify targets or find possible binding modes of the compound against its biological target, and has been successfully used to investigate binding modes of many classes of pesticides [44,45].

Therefore, our interest now focused on the modification of scopoletin to increase its acaricidal potency by using a molecular hybridization method. A series of scopoletin phenolic ether derivatives were designed and synthesized. All the target compounds were characterized by IR, ^1^H-NMR, ^13^C-NMR, MS, and elemental analysis, and their acaricidal activities against female adults of *T. cinnabarlnus* were evaluated. QSAR and molecular docking were also performed to provide useful structure-activity relationship information for the discovery of novel acaricidal agents and insights into the important interaction of compounds and *TcPMCA1*. An in silico study of scopoletin and its synthetic phenolic ether derivatives was performed to predict their ADME properties.

## 2. Results and Discussion

### 2.1. Chemistry

**Scheme 1:**
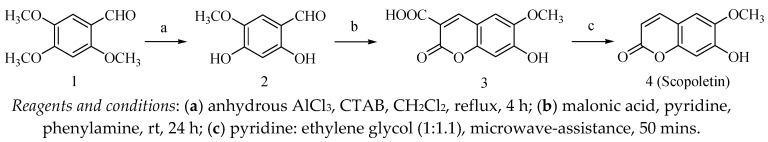
Synthesis of scopoletin.

The synthesis of scopoletin (**4**) is outlined in Scheme 1. The first two reaction steps of synthesizing **4** (scopoletin) followed literature methods [22,46], and the last step was altered by adding the 7-hydroxy-6-methoxy-2-oxo-2*H*-chromene-3-carboxylic acid (**3**) to pyridine and ethylene glycol (1:1.1) and heating under microwave irradiation for 50 min to afford scopoletin (**4**). 

**Scheme 2:**
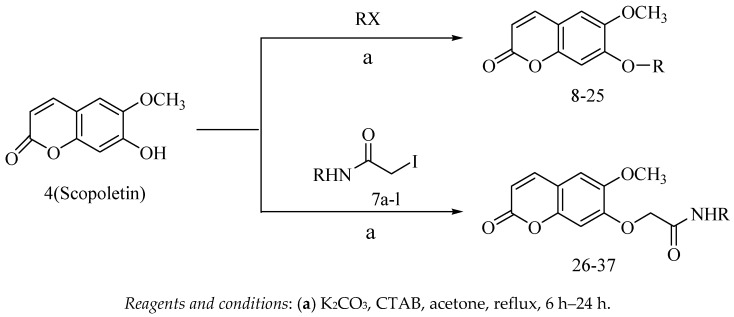
Synthesis of scopoletin phenolic ether derivatives (**8**–**37**).

The synthesis of target compounds **8**–**37** is outlined in Scheme 2. Target compounds **8**–**25** were obtained from scopoletin through a one-step reaction with alkyl or aromatic halides. To increase the electronegativity of the oxygen atom of the hydroxyl from scopoletin, we first added scopoletin and K_2_CO_3_ to acetone and stirred at reflux; subsequently alkyl or aromatic halides were added into the mixed reaction solution to react, leading to the acceptable yields of the derivatives. 

**Scheme 3:**
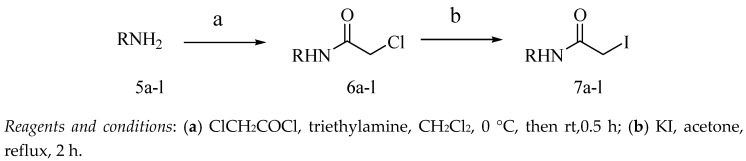
Synthesis of iodoacetamide derivatives.

Target compounds **26**–**37** were obtained through a three-step reaction. The intermediates **6a**–**l** were synthesized through the reaction of 2-chloroacetyl chloride with an alkylamine or substituted benzylamine. To quickly remove the by-product hydrochloric acid from this reaction, we used triethylamine as acid-binding agent, which was added before adding 2-chloroacetyl chloride into the reaction mixture. Chlorinated intermediates **6a**–**l** were converted into iodine-substituted intermediates **7a**–**l** (Scheme 3) to obtain high yields of the target compounds. Finally, the target compounds **26**–**37** were synthesized by reacting scopoletin with intermediates **7a**–**l** in acetone. All of the target compounds provided satisfactory analytical and spectroscopic data, which were consistent with their depicted structures.

### 2.2. Acaricidal Activity

As shown in Table 1, all the tested compounds exhibited varying degrees of acaricidal potency against female adults of *T. cinnabarinus* after treatment for 48 h, LC_50_ (mmol/L) and χ^2^ values of all tested compounds are less than 6.6 and 5.6, respectively, pLC_50_ (mol/L) and *P* values of all tested compounds are more than 2.0 and 0.1, respectively. Except for compound **15**, the rest of the target compounds exhibited more pronounced acaricidal activities against *T. cinnabarinus* than scopoletin. In particular, compounds **32**, **20**, **28**, **27** and **8** exhibited about 8.41-, 7.32-, 7.23-, 6.76-, and 6.65-fold higher acaricidal potency than the lead compound. Compound **32** possessed the the most promising acaricidal activity and exhibited about 1.45-fold higher acaricidal potency against *T. cinnabarinus* than propargite.

The acaricidal activity of compounds **8**–**13** decreased as the carbon chain length of the substituent groups increased. Compounds **14**–**17** with a naphthenic base displayed lower acaricidal activity. When the hydrogen of a hydroxyl or amino from compounds **18**–**25** and **32**–**37** was substituted by a substituted phenyl group, different acaricidal potency was shown. This is due to the types, quantity and position of substituents on the benzene rings of these compounds. However, all of them well followed the Topliss tree rule [47]. Compound **23** with a *para*-chlorinated phenyl (pLC_50_ = 2.3759) exhibited low potency against *T. cinnabarinus* compared with compound **18** with a simple phenyl (pLC_50_ = 2.5922), therefore, both compound **19** with *para*-methylphenyl (pLC_50_ = 2.4954) and compound **22** with 3,4 dichlophenyl (pLC_50_ = 2.7821) don’t show excellent potency against *T. cinnabarinus*. Compound **33** with a *para*-chlorinated phenyl containing an amide group (pLC_50_ = 3.0716) exhibited equivalent potency against *T. cinnabarinus* compared with compound **31** with a phenyl-containing amide group (pLC_50_ = 2.9452), compound **35** with a *para*-methylphenyl-containing amide group exhibited low potency against *T. cinnabarinus* (pLC_50_ = 2.7510) compared with compound **33**, therefore, the compound **32** with a 3-chlorophenyl-containing amide group (pLC_50_ = 3.1835) show higher acaricidal potency. Both compound **34** with a 3,4-dichlorophenyl-containing amide group (pLC_50_ = 3.0165) and compound **36** with a *para*-methoxyphenyl-containing amide group (pLC_50_ = 2.8193) don’t show good potency against *T. cinnabarinus*.

The amino hydrogens of compounds **26**–**31** were substituted by alkyl groups, which also followed the Topliss tree rule [47]. Compound **29** with isopropyl (pLC_50_ = 2.5165) show equivalent potency against *T. cinnabarinus* compared with compound **26** with methyl (pLC_50_ = 2.5950), and compound **27** with an ethyl moiety shows prominent (pLC_50_ = 3.0888) acaricidal potency.

In addition, the acaricidal activity of the compounds **32**–**34** containing amide groups with benzene rings substituted by electron-withdrawing groups (3-chloro-, 4-chloro-, and 3,4-dichloro-) was higher than that of benzene rings substituted by electron-donating groups (4-methyl-, 4-methoxy- and 4-*tert*-butyl-) (compounds **35**–**37**).

The 48 h LC_50_ value of scopoletin in the current study was different from our previous reports [23], which may be attributed to the differences in scopoletin purity, the pesticide adjuvants, and the solvents used to prepare the tested compounds. 

Compound **8** was used as typical representative of all target compounds to evaluate acaricidal activity against eggs, larval, and nymphal of *T. cinnabarinus*, basing on its higher acaricidal potency against female adults. As shown in Table 2, compound **8** exhibits excellent acaricidal potency against larva, low activity against nymphs, and no ovicidal activity. The different acaricidal potency maybe due to different expression of the possible target gene *TcPMCA1* at different stages of *T. cinnabarinus* [36].

### 2.3. QSAR Analysis

#### 2.3.1. 2D-QSAR Analysis

The selected descriptors, their correlations and their values of the investigated compounds are provided in Table 3, Table 4 and Table 5, respectively. The best performing 2D-QSAR models was successfully constructed as shown in Equation (1):

pLC_50_ = 4.243 (± 0.704) − 1.045 (± 0.218) R8e + 12.920 (± 1.921) HATS5p + 0.313 (± 0.062) Depressant − 80 − 1.351 (±0.385) MATS6e − 1.274 (± 0.401) HNar (1)

N = 25, *n* =5, R = 0.935, R^2^_train_ = 0.875, R^2^_adjusted_ = 0.842, RMSE_train_ = 0.1095, F = 26.527 > F_0.005(5,25)_ = 4.43 (the cut off value of F distribution)

R _LOO_ = 0.876, R^2^_LOO_ = 0.768, R^2^_LOO adjusted_ = 0.758, and RMSE _LOO_ = 0.1299, F = 75.998, R^2^_pred_ = 0.583

The R^2^_train_ value of this model reveals that it can explain 87.5% of the variances in activity. Root mean square error (RMSE_train_ = 0.1095) is also a measurable value for the attained model together with the Fisher test value (F = 26.527) which reflects the ratio of the variance explained by the model and the variance due to their errors. A high value of F-test compared with the RMSE is a validation of the model.

To determine whether multicollinearity existed among the descriptors in the models or not, a variable inflation factor (VIF) (VIF = 1/(1 − Rj2), where Rj2 represents the multiple correlation coefficient of one descriptor’s effect on the remaining molecular descriptors) was calculated for each variable in the regression equation [48]. If VIF ranges from 1.0 to 5.0, the linked equation is suitable [49]. As shown in Table 3, the VIF of all descriptors were smaller than 2, indicating that the generated model possessed statistical significance and good stability. Table 4 gives the correlation matrix of the selected descriptors. From this table, it can be seen that the linear correlation coefficient value for each pair of descriptors was smaller than 0.6, suggesting that the selected descriptors were independent, meeting the important criterion for the model selections [50].

**Figure 1:**
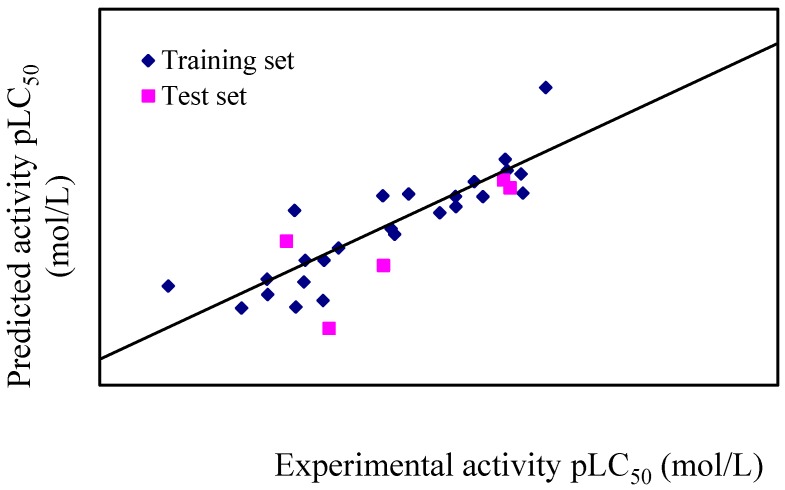
Plot of experimental versus predicted pLC_50_ (mol/L) values of training sets and test sets against female *T. cinnabarinus*.

The reliability and statistical relevance of the attained BMLR-QSAR model is examined by internal and external validation procedures. Experimental and predicted activities (pLC_50_, mol/L) values of the compounds are shown in Table 5 and Figure 1. The residual values obtained by calculating the difference between the predicted and experimental pLC_50_ are below 0.35 logarithmic units for all the compounds.

Internal validation is applied by the SPSS technique employing Leave One Out (LOO), which involves developing a number of models with one example omitted at a time. The observed correlations due to the internal validation techniques are R^2^_LOO_ = 0.768. The R^2^_LOO_ value was bigger than 0.5, indicating that the developed model had good stability and predictive ability [48].

The synthesized thirty target compounds were randomly divided into a 25-molecule training set with LC_50_ values range from 0.655 to 6.588 mmol/L and a 5-molecule (**11**, **16**, **19**, **27**, and **33**) test set with LC_50_ values range from 0.815 to 3.196 mmol/L were used as an external test set for validating the attained QSAR models. The predicted/estimated acaricidal properties of the test set compounds are close to their experimentally observed values preserving their potencies. In addition, the value of R^2^_pred_ = 0.583 for the external prediction was an acceptable result, which conformed that the generated MLR model was useful for meaningful predictions.

The QSAR model indicated that the descriptors representing polarizability (HATS5p) is main property governing acaricidal active agent of the scopoletin phenolic ether derivatives as shown by its high regression coefficient values of 12.920 (Equation (1)). The QSAR model demonstrated that high values of HATS5p, and Depressant-80, but low value of R8e, MATS6e, and HNar are required for potent activity of the compounds. Among these compounds **8**–**25**, compounds **8** and **9** substituted by methyl and ethyl possessed high polarizability (HATS5p: 0.118 and 0.146) and low electronegativity (R8e: 0.384 and 0.706) exhibited high acaricidal potency. Among compounds **25**–**37**, compounds **28** and **32** containing an amide group substituted by *n*-propyl and 3-Cl-benzyl possessed high polarizability (HATS5p: 0.115 and 0.133) and low electronegativity (R8e: 0.609 and 0.567; MATS6e: 0.037 and −0.063) and also displayed high acaricidal potency.

#### 2.3.2. 3D-QSAR Analysis

Table 6 shows the PLS results of the CoMFA and CoMSIA models. The results showed that the optimal CoMFA model yielded a cross-validated q^2^ = 0.802 with an optimal number of principal components (ONC) of 6, non-cross-validated R^2^ of 0.993, SEE = 0.029 and F value of 422.047. The contribution of steric and electrostatic fields is 70.8% and 29.2%, respectively. The best CoMSIA model yielded a q^2^ of 0.735 with an ONC of 6, non-cross-validated R^2^ of 0.965, SEE = 0.059 and F value of 83.553. The contribution of steric, electrostatic, hydrophobic, and hydrogen-bond acceptor are 21.5%, 28.5%, 44.9%, and 5.0%, respectively. Based on these field contributions, the steric field is the most important field in the CoMFA model, whereas the hydrophobic field is the most important field in the CoMSIA model. All the parameters in the Table 6 indicate that the CoMFA and CoMSIA models are robust and stable. 

**Figure 2:**
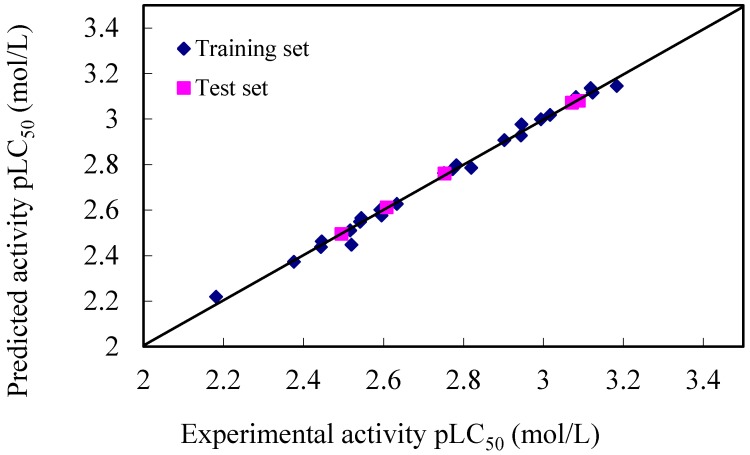
The plot of experimental versus calculated pLC_50_ values from CoMFA analyses for the training and test set compounds.

**Figure 3:**
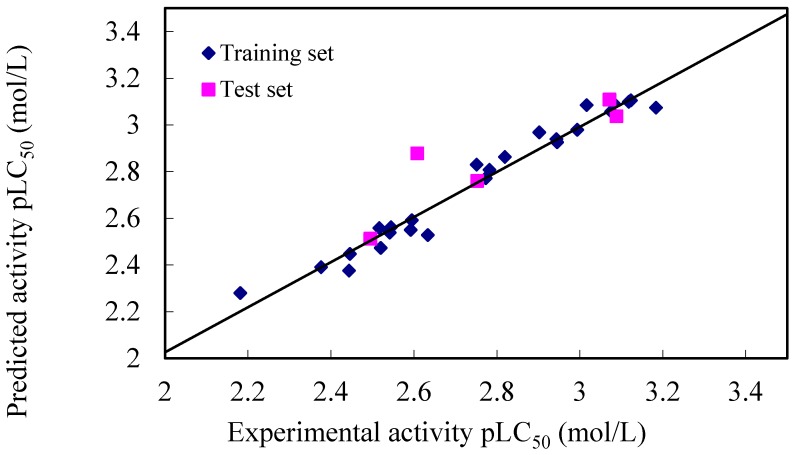
The plot of experimental versus calculated pLC_50_ values from CoMSIA analyses for the training and test set compounds.

The plot of experimental versus predicted acaricidal activities for CoMFA and CoMSIA models are shown in Table 7, and Figure 2 and Figure 3. The residual values obtained by calculating the difference between the predicted and experimental pLC_50_ are below 0.3 logarithmic unit for all the compounds. In addition, the values of R^2^_pred_ = 0.999 (CoMFA) and R^2^_pred_ = 0.787 (CoMSIA) for the external prediction were acceptable results. The CoMFA R^2^_pred_ is higher than its R^2^, which indicated CoMFA model higher predictive ability. The prediced pLC_50_ values of five test compounds by CoMFA model are very close to their experimental pLC_50_ values, and their residuals are less than 0.008 logarithmic unit. These results indicate that the CoMFA and CoMSIA models are predictive. 

**Figure 4:**
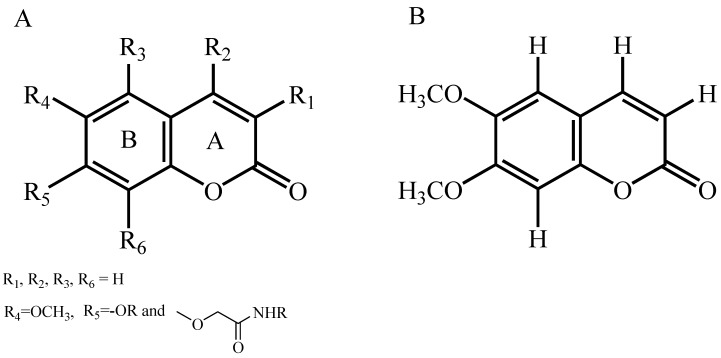
(**A**) The skeleton structure of the studied scopoletin phenolic ether derivatives and (**B**) the chemical structure of compound **8**.

Core structure of the studied scopoletin phenolic ether derivatives were shown in Figure 4A, the compound **8** was employed as the template molecule for the analysis of contour maps (Figure 4B).

**Figure 5:**
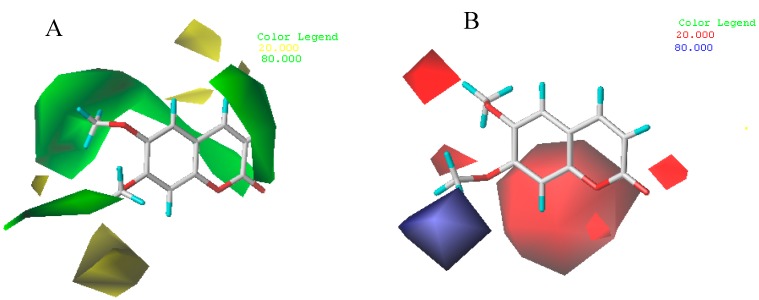
CoMFA STDEV*COEFF contour maps around the compound **8**. (**A**) Steric fields: green contours indicate regions where bulky groups increase activity, while yellow contours indicate regions where bulky groups decrease activity; and (**B**) electrostatic fields: blue contours indicate regions where electron positive groups increase activity, while red contours indicate regions where electron negative groups increase activity. Compound **8** is displayed as a reference.

The CoMFA steric and electrostatic contour maps are shown in Figure 5 with compound **8**. Those contours depict default contribution levels. In the CoMFA steric field shown in Figure 5A, A large-sized and two medium-sized green contour near R_4_, R_1_, R_2_ and R_5_ (C_6_, C_3_, C_4_, and C_7_) indicate that bulky substituents were preferred here. It can be explained that the most of synthesized 7-position scopoletin phenolic ether derivatives have higher acaricidal activity than scopoletin. For CoMFA electrostatic map (Figure 5B), there is one blue contour around the R_5_ (C_7_) position, which can be explain the fact that compound **8** with the smallest electronegative OCH_3_ groups possesses higher acaricidal activity among the synthesized target compounds.

**Figure 6:**
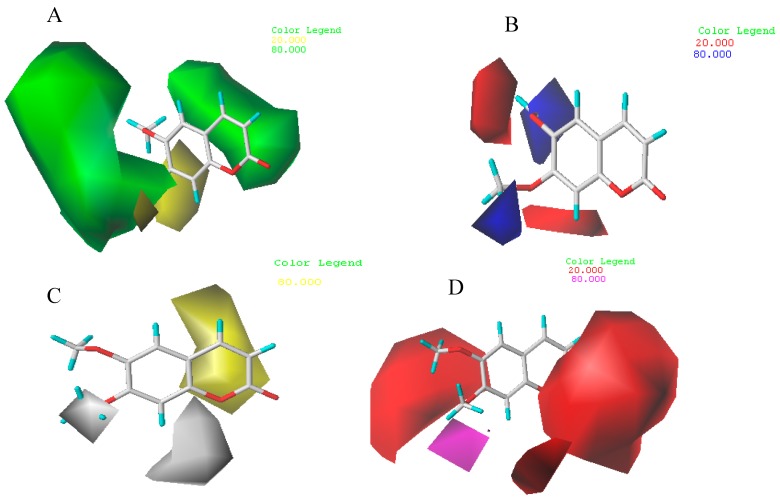
CoMSIA STDEV*COEFF contour maps around the compound **8**. (**A**) Steric fields: green contours and yellow contours show regions where an increase in bulky groups will increase and decrease activity, respectively; (**B**) electrostatic fields: blue contours and red contours show regions where an increase in electron positive groups and electron negative groups will increase activity, respectively; (**C**) hydrophobic fields: yellow contours and white contours show regions where an increase in lipophilicity and hydrophilicity will increase activity, respectively; (**D**) H-bond acceptor contour map: magenta contour and red contour show regions where an increase in hydrogen-bond acceptor groups and hydrogen-bond donor groups will increase activity.

CoMSIA steric, electrostatic, hydrophobic, hydrogen-bond acceptor field contour maps are shown in Figure 6 with compound **8** as an example. Those contours also depict the default contribution levels. Since the steric and electrostatic contour are very similar with that of CoMFA, only hydrophobic, hydrogen-bond acceptor will be described as follows: in the hydrophobic contour map (Figure 6C), a large-sized yellow contour near R_1_ (C_3_) and R_2_ (C_4_) indicates that introducing hydrophobic groups to that position could increase the acaricidal activity of the molecule. A large-sized white contour near R_6_ (C_8_) suggests that hydrophilic substitutes preferentially localize at these positions. A medium-sized white contour were found surrounding the R_5_ (C_7_) which indicates that introducing hydrophilic groups to this position could improve the acaricidal activity. Therefore, the synthesized target compouds (e.g., **27**, **28** and **32**) containing amide groups show higher acaricidal activity.

In H-bond acceptor contour maps (Figure 6D), two large-sized red contours near R_1_ (C_3_) and R_4_ (C_6_) indicate that introducing H-bond donors groups at those positions could increase the acaricidal activity. A medium–sized magenta contour near R_5_ (C_7_) suggests that H-bond acceptor groups at this position are favorable, and will increase the molecular activity. For example, several compounds (e.g., **8**, **9** and **12**) with H-bond acceptor groups display higher acaricidal activity.

In this research, only R_5_ (C_7_)-position was be modified to investigate acaricidal activity, and the contours maps of CoMFA and CoMSIA-derived models suggest that some favored group introduced to other position of scopoletin could improve acaricidal activity, which need to be further study.

### 2.4. Molecular Docking

Some tested compounds exhibit higher acaricidal activity than scopoletin, which prompted us to performed molecular docking study to understand the ligand-the target protein Ca^2+^-ATPase interactions in detail. Scopoletin and the synthesized derivatives **8**, **9**, **12**, **20** and **28** possessing the higher acaricidal activity were selected for the docking study. 

**Figure 7:**
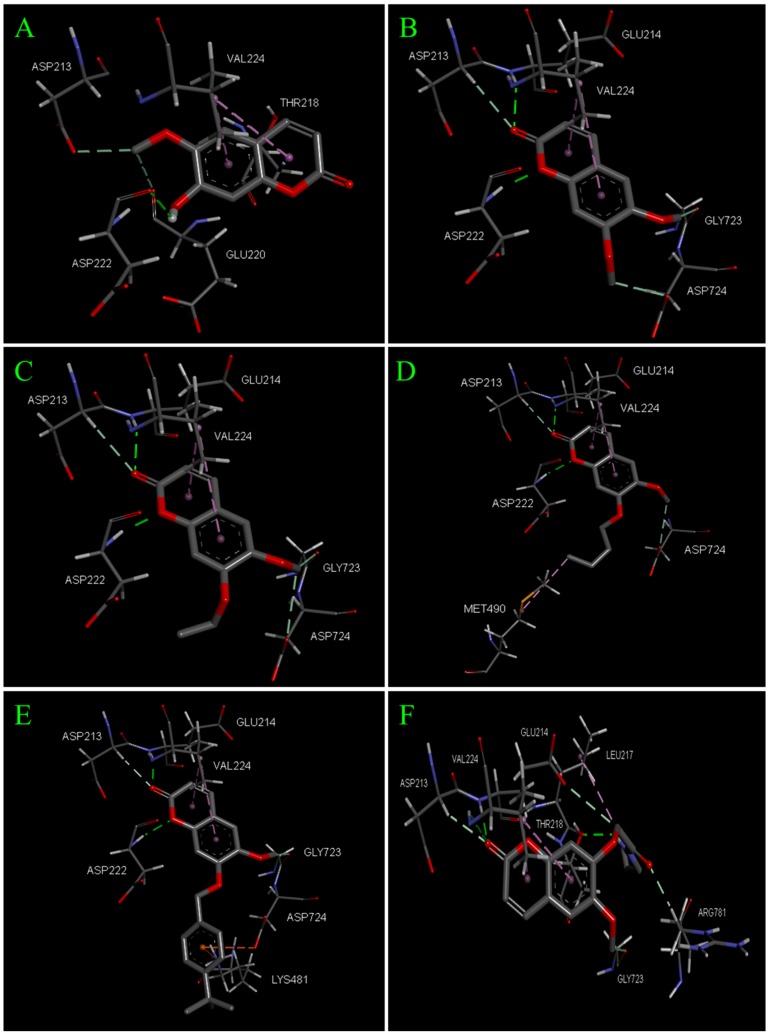
The recognized binding modes and molecular interactions of the scopoletin (**A**) and compounds **8** (**B**), **9** (**C**), **12** (**D**), **20** (**E**), **28** (**F**) in the active site of *TcPMCA1*.

**Figure 8:**
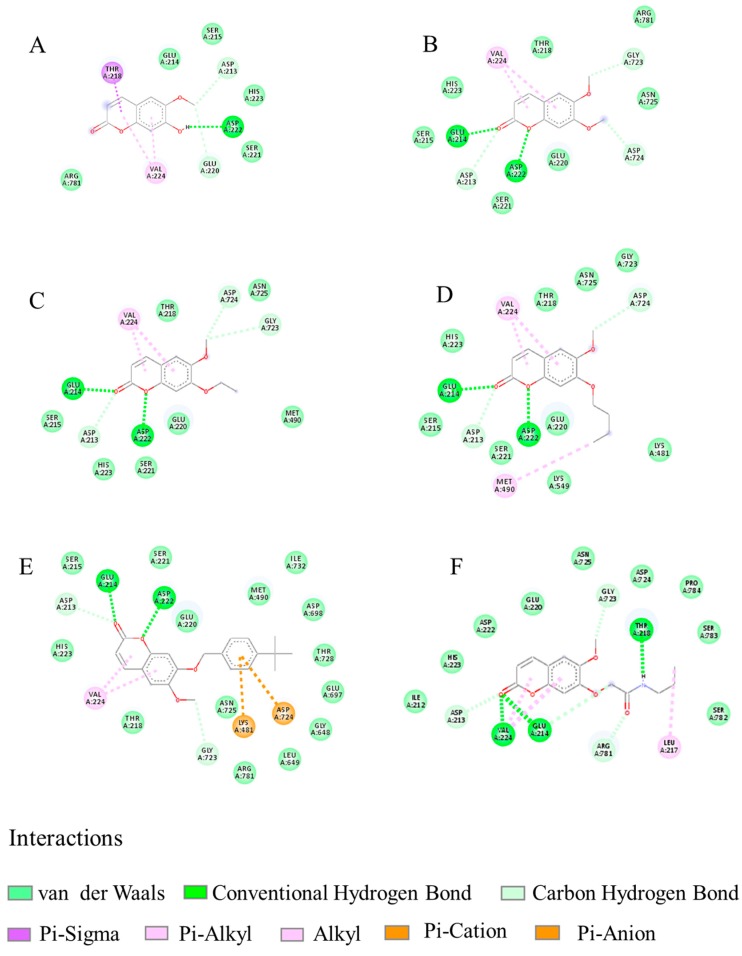
Two dimensional interactions scheme of scopoletin (**A**) and compounds **8** (**B**), **9** (**C**), **12** (**D**), **20** (**E**), and **28** (**F**) to *TcPMCA1*.

Docking results of scopoletin and its derivatives **8**, **9**, **12**, **20** and **28** binding to *TcPMCA1* are listed in Table 8. Scopoletin and selected compound show low binding energies of much less than 5.0 kcal/mol, which can be generally considered as specific ligands of *TcPMCA1*. Figure 7 shows the binding modes and orientations of scopoletin and its derivatives to *TcPMCA1*. The two dimensional interaction diagrams of scopoletin and its derivatives to *TcPMCA1* are shown in Figure 8. Scopoletin exhibits different binding poses compared to its derivatives. Five key amino acids (ASP222, ASP213, GLU220, VAL224, and THR218) in the binding pocket interact with scopoletin via hydrogen bonding and hydrophobic interaction. The H atom of hydroxyl at the 7-position in ring B forms a conventional hydrogen bond (-H…OC-, 2.05 Å) with ASP222, the H atom of methoxy group at the 6-position in ring B forms two nonconventional hydrogen bond (-H…OC-, 2.81 Å; -H…OC-, 3.33 Å) with ASP213, and GLU220 respectively. The benzene and furan rings of scopoletin formed two pi–alkyl (4.07 Å and 4.88 Å) interactions with the VAL224, and furan ring of scopoletin formed a pi–sigma (2.23 Å) interaction with the THR218. In addition, acid residues GLU214, SER215, HIS223, SER221, and ARG781 in the binding pocket interact with scopoletin via Van der Waals interactions. Derivatives **8**, **9**, **12**, and **20** display almost same binding mode, especially homologues **8**, **9** and **12**. The interactions of these compounds with *TcPMCA1* are analyzed using compound **8** as an example. Six key amino acids (GLU214, ASP222, ASP213, GLY723, ASP724, and VAL224) in the binding pocket interact with compound **8** via hydrogen bonding and hydrophobic interaction. The O atom of carbonyl at the 2-position in ring A and O atom of furan ring form two conventional hydrogen bond (-O…NH-, 2.15 Å and -O…NH-, 1.97 Å) with GLU214, and ASP222, respectively. The H atom of the methoxy group at the 6,7-position in ring B and the O atom of carbonyl at the 2-position in ring A form three non-conventional hydrogen bonds (-H…OC-, 3.31, -H…OC-, 3.11, and -O…HC) with GLY723, ASP724, and ASP213, respectively. The furan and benzene rings of compound **8** form two pi–alkyl (4.53 Å and 5.47 Å) interactions with the VAL224. Compound **8** is also surrounded by SER215, HIS223, THR218, ARG781, ASN725, GLU220, and SER221 through Van der Waals interactions. There are few differences of specific biding poses between compounds **8**, **9**, **12** and **20**. Compound **9** shows an analogous binding mode, except for some differences of binding bond length and two non-conventional hydrogen bonds only form at 6-position instead of the 6,7-positions, compared to compound **8**. Compound **12** forms an alkyl–alkyl hydrophobic interaction at the 7-position with MET490 instead of a non-conventional hydrogen bond, compared to compound **8**. Compound **20** forms pi–cation and pi–anion interactions with LYS481, ASP724, respectively, instead of non-conventional hydrogen bonds, compared to compound **8**. Compound **28** with an amide group displays very different binding modes from compounds **8**, **9**, **12** and **20**. Seven key amino acids (GLU214, VAL224, THR218, ASP213, GLY723, ARG781, and LEU217) in the binding pocket interact with compound **28** via hydrogen bonding and hydrophobic interactions. The O atom of the carbonyl at the 2-position in ring A, and the H atom of the amino group at the 7-position in ring B form three conventional hydrogen bonds (-O…NH-, 1.77 Å, -O…NH-, 2.96 Å, -H…OC-, 2.15 Å) with GLU214, VAL224, and THR218, respectively. The O atom of the carbonyl at the 2-position in ring A, the H atoms of the methoxy group at the 6-position, the O atom of the acylamino at the 7-position, the H atom of the methylene at the 7-position in ring B form three non-conventional hydrogen bonds (-O…HC-, 2.53 Å, -H…OC-, 3.16 Å, -O…CH-, 2.21 Å, and -H…OC-,3.53 Å) with ASP 213, GLY 723, ARG 781, and GLU 214, respectively. The furan and benzene rings of compound **28** form two pi–alkyl (3.78 Å and 4.45 Å) interactions with the VAL224. The N-propyl connected to the amide at the 7-position forms alkyl–alkyl interactions with the LEU217. Compound **28** interacts with ILE212, HIS223, ASP222, GLU220, ASN725, ASP724, PRO784, SER783, and SER782 through Van der Waals interactions. These selected derivatives display higher acaricidal activity that may be due to their different binding modes with *TcPMCA1* from the lead compound and they interact with more key amino acid residues. Three selected homologues bind tighter with shortening of the side chain at the 7-position. The binding modes of scopoletin with *TcPMCA1* in the current study were different from our previous reports [32], which may be attributed to our use of different software to find the active binding site interactions of *TcPMCA1*.

### 2.5. ADME Study

An in silico study of scopoletin and its semisynthesed derivatives (compounds **8**–**37**) was performed for prediction of ADME properties [51] (Table 9). From all these parameters, it can be observed that all tested compounds exhibited excellent % absorption (76.40–92.21%). It was also observed that all of these compounds followed Lipinski’s rule of five and its extensions well. Four typical Lipinski’s rule criteria are logP (octanol–water partition coefficient) ≤5, molecular weight ≤500, number of hydrogen bond acceptors ≤10 and number of hydrogen bond donors ≤5. Extension of Lipinski’s rule of five includes the following criteria: number of rotatable bonds ≤10, topological polar surface area ≤140 A^2^.

## 3. Materials and Methods

### 3.1. General Information

Microwave-assisted synthesis was performed on a CW-2000 Ultrasonic Microwave Assisted Extractor (Xintuo Analytical Instruments Co., Ltd., Shanghai, China). Melting points were determined on a WRS-1B Digital Melting-Point Apparatus (Shanghai Shenguang Instrument Co., Ltd., Shanghai, China) and were uncorrected. IR spectra were obtained on a TENSOR 27 FT-IR spectrometer (Bruker Spectroscopic Instruments Co., Rheinstetten, Germany) using KBr pellets and values were presented in cm^−1^. ^1^H- and ^13^C-NMR spectra were recorded on an Avance III 400 NMR spectrometer (Bruker Spectroscopic Instruments Co., Rheinstetten, Germany) with CDCl_3_ or DMSO-*d*_6_ as solvent. Mass spectra were carried out with a GCMS-QP2010 Ultra instrument (Shimadzu Corporation, Kyoto, Japan). Elemental analyses were performed on a Vario EL III elemental analyzer (Elementar Analysensysteme GmbH, Hanau, Germany)Propargite 90.00% TC was provided by Qingdao Hansen Biologic Science Co., Ltd., (Qingdao, China), and all other chemicals and solvents were of analytical grade and used as purchased. Analytical thin-layer chromatography (TLC) was performed on a glass plate coated with silica gel GF-254 (Qingdao Haiyang Chemical Co., Ltd., Qingdao, China) and visualized under ZF-1 ultraviolet analyzer (Shanghai Gucun Electro-optical Instrument Factory, Shanghai, China) under UV light (254 nm). Column chromatography was performed on silica gel (200 to 300 mesh). 

### 3.2. Chemistry

#### 3.2.1. Procedure for the Synthesis of Scopoletin (**4**)

Preparation of 2,4-dihydroxy-5-methoxybenzaldehyde (**2**)

Following a literature method [22,46], aluminum (III) chloride (40 g, 0.30 mol) and CTAB (3.2 g, 8.8% mol) were added into dichloromethane (400 mL), and the reaction mixture was stirred at room temperature for 30 min, then a solution of 2,4,5-trimethoxybenzaldehyde (**1**, 20 g, 0.1 mol) in dichloromethane (100 mL) was added dropwise, then the mixture was refluxed for 4 h (the reaction progress was monitored by TLC with UV detection). The reaction mixture was cooled and poured onto 500 g of ice to which 100 mL of concentrated hydrochloric acid was added. The organic layer was separated and was washed with saturation salt solution, dried over anhydrous sodium sulfate, evaporated under reduced pressure to give 2,4-dihydroxy-5-methoxybenzaldehyde (**2**) as a light yellow solid, 60.84% yeild, m.p. 152–153 °C.

Preparation 7-hydroxy-6-methoxy-2-oxo-2*H*-chromene-3-carboxylic acid (**3**)

2,4-Dihydroxy-5-methoxybenzaldehyde (**2**, 9.49 g, 56 mmol), malonic acid (13.5 g, 130 mmol), and phenylamine (1 mL) were added into pyridine (30 mL), the resulting solution was stirred at room temperature for over 24 h and then acidified to pH 4 using dilute HCl. The precipitate was collected by suction filtration and followed by recrystallization from ethanol to give 7-Hydroxy-6-methoxy-2-oxo-2*H*-chromene-3-carboxylic acid (**3**) as a yellow solid, 80.01% yeild, m.p. 231–232 °C.

Preparation of 7-hydroxy-6-methoxy-2*H*-chromen-2-one (scopoletin, **4**)

7-Hydroxy-6-methoxy-2-oxo-2*H*-chromene-3-carboxylic acid (**3**, 3.5 g, 14.8 mmol) was refluxed in ethylene glycol (17.6 mL) and pyridine (16 mL) for 50 min under microwave irradiation (115 W) and keep temperature above 110 °C. After cooling the reaction, the mixture was acidified to about pH 5 using a solution of diluted HCl 30 mL, then 7-hydroxy-6-methoxy-2*H*-chromen-2-one (scopoletin, **4**) crystals were obtained after standing overnight, the filter liquor was extracted with CH_2_Cl_2_. The CH_2_Cl_2_ layers were pooled and washed with saturation sodium bicarbonate, and saturation salt solution, successively, dried over anhydrous sodium sulfate, evaporated under reduced pressure and followed by recrystallization from acetone to give the target product **4**, 65.74% total yield, m.p. 201–202 °C.

#### 3.2.2. General Procedure for the Synthesis of **8**–**13** and **18**–**25**

K_2_CO_3_ (0.2073 g, 3 mmol) and CTAB (54.67 mg, 7.5% mmol) were added into a solution of scopoletin (**4**, 0.3843 g, 2 mmol) in acetone (30 mL), and the reaction mixture was stirred at reflux for 30 min. Then an alkyl or aromatic halide (3 mmol) was added into the mixture and maintained at reflux for 6–24 h (the reaction progress was monitored by TLC with UV detection). After cooling the reaction and filtration, the solvent was evaporated under reduced pressure, and the residue was dissolved in ethyl acetate, washed with saturation sodium bicarbonate, and saturation salt solution, successively, dried over anhydrous sodium sulfate, evaporated under reduced pressure to give the target crude products. The crude products were purified by column chromatography using petroleum ether/ethyl acetate from 10:1 to 7:1 as the gradient eluent system to yield the products **8**–**13** and **18**–**25**.

6,7-Dimethoxy-2*H*-chromen-2-one (**8**) 

White needle-like crystals; 70.83% yield; m.p. 145.2–146.9 °C; IR *ν*_max_ (KBr) cm^−1^: 3082, 2938, 2877, 1713, 1619, 1559, 1513, 1467, 1424, 1379, 1275, 1248, 1169, 1027, 880; ^1^H-NMR (DMSO-*d*_6_, δ ppm): 3.81 (s, 6H, 2 × OCH_3_), 6.30 (d, 1H, *J* = 8Hz, C_3_-H), 7.05 (s, 1H, C_8_-H), 7.25 (s, 1H, C_5_-H), 7.96 (d, 1H, *J* = 12 Hz, C_4_-H); ^13^C-NMR (DMSO-*d*_6_, δ ppm): 56.31, 70.56, 100.98, 109.39, 111.51, 112.99, 144.82, 146.36, 149.85, 152.32, 161.07; MS (*m*/*z*): [M]^+^ 206. Anal. Calcd. for C_11_H_10_O_4_: C, 64.08%; H, 4.89%. Found: C, 64.22%; H, 4.94%.

7-Ethoxy-6-methoxy-2*H*-chromen-2-one (**9**) 

White granular crystals; 75.02% yield; m.p. 146.3–146.7 °C; IR *ν*_max_ (KBr) cm^−1^: 3064, 2982, 2916, 2882, 2830, 1705, 1616, 1600, 1510, 1465, 1424, 1388, 1274, 1247, 1147, 1023, 877. ^1^H-NMR (DMSO-*d*_6_, δ ppm): 1.37 (t, 3H, *J* = 8 Hz, CH_3_), 3.81 (s, 3H, OCH_3_), 4.12 (q, 2H, *J* = 8 Hz, CH_2_), 6.30 (d, 1H, *J* = 12 Hz, C_3_-H), 7.04 (s, 1H, C_8_-H), 7.24 (s, 1H, C_5_-H), 7.95 (d, 1H, *J* = 8 Hz, C_4_-H) (Figure S1). ^13^C-NMR (DMSO-*d*_6_, δ ppm): 14.87, 56.24, 64.86, 100.95, 109.30, 111.51, 113.00, 144.81, 146.30, 149.84, 152.17, 161.06 (Figure S2). MS (*m*/*z*): [M]^+^ 220. Anal. Calcd. for C_12_H_12_O_4_: C, 65.45%; H, 5.49%. Found: C, 65.62%; H, 5.52%.

6-Methoxy-7-propoxy-2*H*-chromen-2-one (**10**)

White granular crystals; 78.08% yield; m.p. 96.0–96.7 °C, IR *ν*_max_ (KBr) cm^−1^: 3084, 2967, 2939, 2877, 1705, 1619, 1559, 1513, 1467, 1424, 1386, 1275, 1248, 1150, 1027, 880. ^1^H-NMR (DMSO-*d*_6_, δ ppm): 0.99 (t, 3H, *J* = 8Hz, CH_3_), 1.73–1.82 (m, 2H, C′_2_-CH_2_), 3.82 (s, 3H, OCH_3_), 4.02 (t, 2H, *J* = 8 Hz, C′_1_-CH_2_), 6.30 (d, 1H, *J* = 12 Hz, C_3_-H), 7.04 (s, 1H, C_8_-H), 7.24 (s, 1H, C_5_-H), 7.95 (d, 1H, *J* = 8 Hz, C_4_-H) (Figure S3). ^13^C-NMR (DMSO-*d*_6_, δ ppm): 10.81, 22.26, 56.33, 70.57, 100.98, 109.42, 111.51, 112.98, 144.78, 146.38, 149.85, 152.34, 161.05 (Figure S4). MS (*m*/*z*): [M]^+^ 234. Anal. Calcd. for C_13_H_14_O_4_: C, 66.67%; H, 5.98%. Found: C, 66.99%; H, 6.08%.

7-Isopropoxy-6-methoxy-2*H*-chromen-2-one (**11**)

White needle crystals; 58.73% yield; m.p.108.4–108.5 °C; IR *ν*_max_ (KBr) cm^−1^: 3098, 2979, 2939, 1706, 1613, 1560, 1513, 1464, 1424, 1383, 1269, 1245, 1148, 1025, 846. ^1^H-NMR (DMSO-*d*_6_, δ ppm): 1.31 (d, 6H, *J* = 8 Hz, CH_3_), 3.80 (s, 3H, OCH_3_), 4.71–4.80 (m, 1H, CH), 6.29 (d, 1H, *J* = 8 Hz, C_3_-H), 7.08 (s, 1H, C_8_-H), 7.25 (s, 1H, C_5_-H), 7.95 (d, 1H, *J* = 8 Hz, C_4_-H) (Figure S5). ^13^C-NMR (DMSO-*d*_6_, δ ppm): 22.03, 56.24, 71.15, 102.02, 109.63, 111.48, 112.96, 144.78, 146.91, 149.84, 151.03, 161.09 (Figure S6). MS (*m*/*z*): [M]^+^ 234. Anal. Calcd. for C_13_H_14_O_4_: C, 66.67%; H, 6.02%. Found: C, 66.82%; H, 6.21%.

7-Butoxy-6-methoxy-2*H*-chromen-2-one (**12**)

White granular crystals; 66.01% yield; m.p. 77.1–78.3 °C, IR *ν*_max_ (KBr) cm^−1^: 3067, 2940, 2867, 1699, 1609, 1557, 1512, 1465, 1423, 1384, 1277, 1247, 1147, 1023, 873. ^1^H-NMR (DMSO-*d*_6_, δ ppm): 0.95 (t, 3H, *J* = 6 Hz, CH_3_), 1.40–1.49 (m, 2H, C′_3_-CH_2_), 1.70–1.79 (m, 2H, C′_2_-CH_2_), 3.81 (s, 3H, OCH_3_), 4.06 (t, 2H, *J* = 6 Hz, C′_1_-CH_2_), 6.29 (d, 1H, *J* = 8 Hz, C_3_-H), 7.05 (s, 1H, C_8_-H), 7.24 (s, 1H, C_5_-H), 7.95 (d, 1H, *J* = 12 Hz, C_4_-H). ^13^C-NMR (DMSO-*d*_6_, δ ppm): 14.11, 19.18, 30.93, 56.32, 68.85, 100.98, 109.40, 111.51, 112.97, 144.77, 146.38, 149.86, 152.37, 161.05. MS (*m*/*z*): [M]^+^ 248. Anal. Calcd. for C_14_H_16_O_4_: C, 67.73%; H, 6.50%. Found: C, 68.05%; H, 6.51%.

7-Isobutoxy-6-methoxy-2*H*-chromen-2-one (**13**)

White granular crystals; 27.85% yield; m.p. 84.1–85.3 °C; IR *ν*_max_ (KBr) cm^−1^: 3096, 2965, 2921, 2876, 1714, 1613, 1561, 1514, 1457, 1425, 1386, 1266, 1248, 1143, 1013, 864. ^1^H-NMR (DMSO-*d*_6_, δ ppm): 1.00 (t, 3H, C′_2_-CH_3_), 1.44 (d, 3H, *J* = 8 Hz, C′_1_-CH_3_), 2.00–2.13 (m, 2H, CH_2_), 3.82 (s, 3H, OCH_3_), 3.83–3.94 (m, 1H, CH), 6.29 (d, 1H, *J* = 8 Hz, C_3_-H), 7.04 (s, 1H, C_8_-H), 7.25 (s, 1H, C_5_-H), 7.95 (d, 1H, *J* = 8 Hz, C_4_-H). ^13^C-NMR (DMSO-*d*_6_, δ ppm): 19.47, 28.02, 56.47, 75.21, 101.12, 109.61, 111.55, 113.02, 144.82, 146.45, 149.88, 152.45, 161.07. MS (*m*/*z*): [M]^+^ 248. Anal. Calcd. for C_14_H_16_O_4_: C, 67.73%; H, 6.50%. Found: C, 67.16%; H, 6.60%.

7-(Benzyloxy)-6-methoxy-2*H*-chromen-2-one (**18**)

White needle-like crystals; 72.41% yield; m.p. 124.6–125.6 °C; IR *ν*_max_ (KBr) cm^−1^: 2959, 1715, 1611, 1561, 1510, 1462, 1427, 1379, 1274, 1247, 1145, 1027, 757. ^1^H-NMR (DMSO-*d*_6_, δ ppm): 3.93 (s, 3H, OCH_3_), 5.23 (s, 2H, CH_2_O), 6.27 (d, 1H, *J* = 8 Hz, C_3_-H), 6.87 (s, 1H, C_8_-H), 7.26 (s, 1H, C_5_-H), 7.33–7.45 (m, 5H, Ar-H), 7.61 (d, 1H, *J* = 8 Hz, C_4_-H) (Figure S9). ^13^C-NMR (DMSO-*d*_6_, δ ppm): 56.47, 71.13, 101.83, 108.36, 111.69, 113.59, 127.31, 128.37, 128.82, 135.63, 143.31, 146.77, 149.75, 151.83, 161.42 (Figure S10). MS (*m*/*z*): [M]^+^ 282. Anal. Calcd. for C_17_H_14_O_4_: C, 72.33%; H, 5.00%. Found: C, 72.07%; H, 4.98%.

6-Methoxy-7-(4-methylbenzyloxy)-2*H*-chromen-2-one (**19**)

White granular crystals; 80.17% yield; m.p. 126.1–127.0 °C; IR *ν*_max_ (KBr) cm^−1^: 3075, 3011, 2944, 2929, 2861, 1705, 1616, 1563, 1518, 1460, 1425, 1392, 1279, 1249, 1145, 1020, 883. ^1^H-NMR (DMSO-*d*_6_, δ ppm): 2.31 (s, 3H, CH_3_), 3.81 (s, 3H, OCH_3_), 5.15 (s, 2H, CH_2_O), 6.31 (d, 1H, *J* = 12Hz, C_3_-H), 7.15 (s, 1H, C_8_-H), 7.21 (s, 1H, C_5_-H), 7.24, 7.36 (dd, 4H, *J* = 8 Hz, Ar-H), 7.95 (d, 1H, *J* = 8 Hz, C_4_-H). ^13^C-NMR (DMSO-*d*_6_, δ ppm): 21.27, 56.32, 70.62, 101.60, 109.47, 111.78, 113.20, 128.69, 129.54, 133.55, 137.95, 144.77, 146.49, 149.65, 151.84, 161.02. MS (*m*/*z*): [M]^+^ 296. Anal. Calcd. for C_18_H_16_O_4_: C, 72.96%; H, 5.44%. Found: C, 72.53%; H, 5.26%.

7-(4-*tert*-Butylbenzyloxy)-6-methoxy-*2H*-chromen-2-one (**20**)

White sheet-like crystals; 83.23% yield; m.p. 149.4–149.7 °C; IR *ν*_max_ (KBr) cm^−1^: 3082, 2948, 2862, 1720, 1610, 1560, 1508, 1460, 1424, 1387, 1272, 1242, 1141, 1015, 858. ^1^H NMR (DMSO-*d*_6_, δ ppm): 1.28 (s, 9H, 3 × CH_3_), 3.81 (s, 3H, OCH_3_), 5.16 (s, 2H, CH_2_O), 6.30 (d, 1H, *J* = 12 Hz, C_3_-H), 7.17 (s, 1H, C_8_-H), 7.27 (s, 1H, C_5_-H), 7.40, 7.43 (dd, 4H, *J* = 8 Hz, Ar-H), 7.95 (d, 1H, *J* = 8 Hz, C_4_-H). ^13^C-NMR (DMSO-*d*_6_, δ ppm): 31.56, 34.80, 56.35, 70.55, 101.56, 109.54, 111.79, 113.21, 125.75, 128.52, 133.60, 144.77, 146.51, 149.69, 151.15, 151.92, 161.00. MS (*m*/*z*): [M]^+^ 338. Anal. Calcd. for C_21_H_22_O_4_: C, 74.54%; H, 6.55%. Found: C, 75.00%; H, 6.54%.

7-(4-Nitrobenzyloxy)-6-methoxy-2*H*-chromen-2-one (**21**)

White powder; 60.42% yield; m.p. 122.3–122.9 °C; IR *ν*_max_ (KBr) cm^−1^: 2946, 1735, 1603, 1562, 1519, 1465, 1427, 1347, 1284, 1251, 1174, 1010, 853. ^1^H-NMR (DMSO-*d*_6_, δ ppm): 3.95 (s, 3H, OCH_3_), 5.30 (s, 2H, CH_2_O), 6.31 (d, 1H, *J* = 12 Hz, C_3_-H), 6.92 (s, 1H, C_8_-H), 7.27 (s, 1H, C_5_-H), 7.63 (d, 1H, *J* = 4Hz, C_4_-H), 7.65 (d, 2H, *J* = 4 Hz, Ar-H), 8.27 (d, 2H, *J* = 8 Hz, Ar-H). ^13^C-NMR (DMSO-*d*_6_, δ ppm): 56.45, 69.74, 101.84, 108.59, 112.26, 114.14, 124.04, 127.66, 143.13, 146.72, 147.82, 149.55, 151.01, 161.13. MS (*m*/*z*): [M]^+^ 327. Anal. Calcd. for C_17_H_13_NO_6_: C, 62.39%; H: 4.00%; N: 4.28%. Found: C, 62.02%; H, 4.15%; N, 4.49%.

7-(3,4-Dichlorobenzyloxy)-6-methoxy-2*H*-chromen-2-one (**22**)

White granular crystals; 53.08% yield; m.p. 151.5–152.3 °C; IR *ν*_max_ (KBr) cm^−1^: 2948, 1698, 1607, 1564, 1509, 1435, 1375, 1274, 1259, 1139, 1018, 860. ^1^H-NMR (DMSO-*d*_6_, δ ppm): 3.95 (s, 3H, OCH_3_), 5.14 (s, 2H, CH_2_O), 6.27 (d, 1H, *J* = 8 Hz, C_3_-H), 6.85 (s, 1H, C_8_-H), 7.27 (s, 1H, C_5_-H), 7.47 (d, 1H, *J* = 8 Hz, C_4_-H), 7.56–7.64 (m, 3H, Ar-H). ^13^C-NMR (DMSO-*d*_6_, δ ppm): 56.42, 69.67, 101.79, 108.48, 112.08, 113.94, 126.54, 129.22, 130.78, 133.02, 135.87, 143.36, 144.04, 146.71, 149.72, 151.21, 161.27. MS (*m*/*z*): [M]^+^ 351. Anal. Calcd. for C_17_H_12_Cl_2_O_4_: C, 58.14%; H: 3.44%. Found: C, 58.95%; H, 3.91%.

7-(4-Chlorobenzyloxy)-6-methoxy-2*H*-chromen-2-one (**23**)

White powder; 76.13% yield; m.p. 171.8–172.3 °C; IR *ν*_max_ (KBr) cm^−1^: 3074, 3014, 2949, 2927, 2875, 1705, 1615, 1562, 1515, 1461, 1426, 1393, 1279, 1249, 1145, 1015, 880. ^1^H-NMR (DMSO-*d*_6_, δ ppm): 3.81 (s, 3H, OCH_3_), 5.21 (s, 2H, CH_2_O), 6.32 (d, 1H, *J* = 8 Hz, C_3_-H), 7.18 (s, 1H, C_8_-H), 7.30 (s, 1H, C_5_-H), 7.48, 7.51 (dd, 4H, *J* = 8 Hz, Ar-H), 7.97 (d, 1H, *J* = 8 Hz, C_4_-H). ^13^C-NMR (DMSO-*d*_6_, δ ppm): 56.38, 69.80, 101.72, 109.60, 111.98, 113.38, 129.04, 130.34, 133.23, 135.69, 144.77, 146.48, 149.60, 151.57, 160.99. MS (*m*/*z*): [M]^+^ 316. Anal. Calcd. for C_17_H_13_ClO_4_: C: 64.47%; H: 4.14%. Found: C, 64.60%; H, 4.18%.

7-(4-(Trifluoromethyl)benzyloxy)-6-methoxy-2*H*-chromen-2-one (**24**)

White needle-like crystals; 83.56% yield; m.p. 192.8–193.3 °C; IR *ν*_max_ (KBr) cm^−1^: 3073, 2938, 1713, 1616, 1565, 1515, 1463, 1427, 1393, 1281, 1250, 1146, 1019, 881. ^1^H-NMR (DMSO-*d*_6_, δ ppm): 3.94 (s, 3H, OCH_3_), 5.26 (s, 2H, CH_2_O), 6.29 (d, 1H, *J* = 8 Hz, C_3_-H), 6.90 (s, 1H, C_8_-H), 7.27 (s, 1H, C_5_-H), 7.56-7.67 (m, 5H, Ar-H and C_4_-H). ^13^C-NMR (DMSO-*d*_6_, δ ppm): 56.47, 70.22, 101.82, 108.53, 112.03, 113.95, 125.77, 125.81, 127.35, 130.39, 139.71, 143.19, 146.75, 149.65, 151.36, 161.23. MS (*m*/*z*): [M]^+^ 350. Anal. Calcd. for C_18_H_13_F_3_O_4_: C, 61.72%; H, 3.74%. Found: C, 62.35%; H, 4.27%.

7-(4-(Trifluoromethoxy)benzyloxy)-6-methoxy-2*H*-chromen-2-one (**25**)

White powder; 84.32% yield; m.p. 127.0–127.2 °C; IR *ν*_max_ (KBr) cm^−1^: 3056, 2960, 1727, 1615, 1565, 1514, 1466, 1426, 1382, 1248, 1147, 1023, 872. ^1^H-NMR (DMSO-*d*_6_, δ ppm): 3.92 (s, 3H, OCH_3_), 5.18 (s, 2H, CH_2_O), 6.27 (d, 1H, *J* = 8 Hz, C_3_-H), 6.89 (s, 1H, C_8_-H), 7.24 (d, 2H, *J* = 8 Hz, Ar-H), 7.28 (s, 1H, C_5_-H), 7.49 (d, 2H, *J* = 8 Hz, Ar-H), 7.62 (d, 1H, *J* = 12Hz, C_4_-H). ^13^C-NMR (DMSO-*d*_6_, δ ppm): 56.40, 70.17, 101.68, 108.48, 111.92, 113.74, 119.16, 121.26, 128.86, 134.40, 143.28, 146.73, 149.11, 149.65, 151.50, 161.28. MS (*m*/*z*): [M]^+^ 366. Anal. Calcd. for C_18_H_13_F_3_O_5_: C, 59.02%; H, 3.58%. Found: C, 59.87%; H, 4.26%.

#### 3.2.3. General Procedure for the Synthesis of **14**–**17**

K_2_CO_3_ (0.2073 g, 3 mmol) were added into a solution of scopoletin (**4**, 0.3843 g, 2 mmol) in DMF (10 mL), and the mixture was reacted under microwave irradiation (80 W power) for 7 min. Then the naphthenic halide (3 mmol) was added into the mixture and reacted under microwave irradiation (120 W power) for 40 min (the reaction progress was monitored by TLC with UV detection). After cooling the reaction and poured into 15 mL water, the mixture liquor was extracted with ethyl acetate. The ethyl acetate layers were pooled and washed with saturation salt solution, dried over anhydrous sodium sulfate, evaporated under reduced pressure to give the target crude products. The crude products were purified by column chromatography using petroleum ether/ethyl acetate from 10:1 to 7:1 as the gradient eluent system to yield the products **14**–**17**.

7-(Cyclopropylmethoxy)-6-methoxy-2*H*-chromen-2-one (**14**)

Brown sheet-like crystals; 74.59% yield; m.p. 149.2–149.6 °C; IR *ν*_max_ (KBr) cm^−1^: 3076, 2963, 2937, 2880, 1715, 1615, 1563, 1462, 1426, 1387, 1276, 1248, 1146, 1024, 880. ^1^H-NMR (DMSO-*d*_6_, δ ppm): 0.39 –0.43 (m, 2H, CH_2_), 0.68–0.73 (m, 2H CH_2_), 1.32–1.42 (m, 1H, CH), 3.91(d, 2H, *J* = 4 Hz, CH_2_), 3.92 (s, 3H, OCH_3_), 6.28 (d, 1H, *J* = 12 Hz, C_3_-H), 6.86 (s, 1H, C_8_-H), 7.27 (s, 1H, C_5_-H), 7.62 (d, 1H, *J* = 8 Hz, C_4_-H). ^13^C-NMR (DMSO-*d*_6_, δ ppm): 3.58, 9.89, 56.42, 74.19, 100.94, 108.23, 111.33, 113.32, 143.39, 146.58, 149.93, 152.35, 161.49; MS (*m*/*z*): [M]^+^ 246. Calcd. for C_14_H_14_O_4_: C, 68.26%; H, 5.69%. Found: C, 68.38%; H, 5.81%.

7-(Cyclopentyloxy)-6-methoxy-2*H*-chromen-2-one (**15**)

White needle-like crystals; 72.89% yield; m.p. 80.7–81.2 °C; IR *ν*_max_ (KBr) cm^−1^: 3075, 2944, 2867, 1718, 1611, 1558, 1511, 1467, 1425, 1387, 1277, 1248, 1144, 1024, 875. ^1^H-NMR (DMSO-*d*_6_, δ ppm): 1.64–1.70 (m, 2H, CH_2_), 1.82–1.87 (m, 2H, CH_2_), 1.89–1.94 (m, 2H, CH_2_), 1.98–2.07 (m, 2H, CH_2_), 3.88 (s, 3H, OCH_3_), 4.79-4.84(m, 1H, CH), 6.27 (d, 1H, *J* = 12 Hz, C_3_-H), 6.84 (s, 1H, C_8_-H), 7.27 (s, 1H, C_5_-H), 7.62 (d, 1H, *J* = 12 Hz, C_4_-H). ^13^C-NMR (DMSO-*d*_6_, δ ppm): 24.23, 32.78, 56.49, 81.03, 102.06, 108.51, 111.07, 113.07, 143.45, 146.99, 149.95, 151.76, 161.60. MS (*m*/*z*): [M]^+^ 260. Calcd. for C_15_H_16_O_4_: C, 69.23%; H, 6.15%. Found: C, 69.32%; H, 6.24%.

7-(Cyclohexyloxy)-6-methoxy-2*H*-chromen-2-one (**16**)

White granular crystals; 12.21% yield; m.p. 141.9–142.0 °C; IR *ν*_max_ (KBr) cm^−1^: 3075, 2944, 2840, 1716, 1608, 1563, 1514, 1456, 1425, 1387, 1282, 1248, 1144, 1024, 873. ^1^H-NMR (DMSO-*d*_6_, δ ppm): 1.26–1.45 (m, 2H, CH_2_), 1.57–1.66 (m, 2H, CH_2_), 1.83–1.88 (m, 2H, CH_2_), 2.06–2.09 (m, 1H, CH), 3.89 (s, 3H, OCH_3_), 6.27 (d, 1H, *J* = 8 Hz, C_3_-H), 6.86 (s, 1H, C_8_-H), 7.27 (s, 1H, C_5_-H),7.61 (d, 1H, *J* = 12 Hz, C_4_-H) (Figure S7). ^13^C-NMR (DMSO-*d*_6_, δ ppm): 23.91, 25.44, 31.50, 56.56, 77.03, 102.35, 108.81, 111.27, 113.21, 143.30, 147.26, 150.00, 151.32, 161.49 (Figure S8). MS (*m*/*z*): [M + H]^+^ 275. Calcd. for C_16_H_18_O_4_: C, 70.07%; H, 6.57%. Found: C, 70.15%; H, 6.67%.

7-(Cyclohexylmethoxy)-6-methoxy-2*H*-chromen-2-one (**17**)

White granular crystals; 30.23% yield; m.p. 144.4–144.7 °C; IR *ν*_max_ (KBr) cm^−1^: 3072, 2938, 2854, 1713, 1614, 1562, 1514, 1464, 1425, 1385, 1279, 1251, 1144, 1034, 882. ^1^H-NMR (DMSO-*d*_6_, δ ppm): 1.02–1.11 (m, 2H, CH_2_), 1.20-1.32 (m, 4H, 2 × CH_2_), 1.70–1.79 (m, 4H, 2 × CH_2_), 1.89-1.97 (m, 1H, CH), 3.86 (d, 2H, *J* = 4 Hz, CH_2_), 3.90 (s, 3H, OCH_3_), 6.27 (d, 1H, *J* = 8 Hz, C_3_-H), 6.85 (s, 1H, C_8_-H), 7.28 (s, 1H, C_5_-H), 7.62 (d, 1H, *J* = 12 Hz, C_4_-H). ^13^C-NMR (DMSO-*d*_6_, δ ppm): 25.66, 26.40, 29.81, 37.16, 56.54, 74.65, 100.80, 108.48, 111.17, 113.16, 143.41, 146.66, 150.04, 152.72, 161.53. MS (*m*/*z*): [M]^+^ 288. Calcd. for C_17_H_20_O_4_: C, 70.83%; H, 6.94%. Found: C, 70.73%; H, 6.99%.

#### 3.2.4. General Procedure for the Synthesis of **26**–**37**

Triethylamine (0.3643 g, 3.6 mmol) was added to a solution of the appropriate alkylamine or substituted benzylamine **5a**–**l** (3 mmol) in dichloromethane (7.5 mL), and the reaction mixture was stirred for 5 min at room temperature, then 2-chloroacetyl chloride (0.3857 g, 3.6 mmol) was added dropwise to this reaction mixture at 0 °C and stirred for 15 min at room temperature. After completion of the reaction, the solvent was evaporated under reduced pressure to afford **6a**–**l**. KI (0.5976 g, 3.6 mmol) and CTAB (98.40 mg, 7.5% mmol) were added to a solution of the crude product **6a**–**l** in acetone (30 mL) and maintained stirring at reflux for 2 h to afford **7a**−**l**. K_2_CO_3_ (0.2073 g, 3 mmol) was added to a solution of scopoletin (0.3843 g, 2 mmol) in acetone (30 mL), and the reaction mixture was stirred at refluxed for 30 min. Then crude intermediates **7a**–**l** were added into the mixture and maintained reflux for 8–12 h (the reaction progress was monitored by TLC with UV detection). After cooling the reaction and filtration, the solvent was evaporated under reduced pressure, and the residue was dissolved in ethyl acetate, washed with saturation sodium bicarbonate, and saturation salt solution successively, dried over anhydrous sodium sulfate, evaporated under reduced pressure to give the target crude products. The crude products were purified by column chromatography using petroleum ether/ethyl acetate from 6:1 to 2:1 as the gradient eluent system to yield the products **26**–**37**.

2-(6-Methoxy-2-oxo-2*H*-chromen-7-yloxy)-*N*-methylacetamide (**26**)

White needle-like crystals; 21.22% yield; m.p. 155.8–156.40 °C; IR *ν*_max_ (KBr) cm^−1^: 3327, 2982, 1750, 1704, 1609, 1566, 1515, 1425, 1394, 1280, 1251, 1144, 1019, 854. ^1^H-NMR (CDCl_3_, δ ppm): 3.83 (s, 3H, CH_3_), 3.94 (s, 3H, OCH_3_), 4.78 (s, 2H, CH_2_O), 6.32 (d, 1H, *J* = 12 Hz, C_3_-H), 6.91 (s, 1H, C_8_-H), 7.29 (s, 1H, C_5_-H), 7.64 (d, 1H, *J* = 8 Hz, C_4_-H). ^13^C-NMR (DMSO-*d*_6_, δ ppm): 26.48, 52.61, 65.82, 101.45, 108.72, 112.56, 114.21, 143.23, 146.53, 149.40, 150.66, 161.21, 168.21. MS (*m*/*z*): [M + H]^+^ 264. Anal. Calcd. for C_13_H_13_NO_5_: C, 59.31%; H, 4.98%; N, 5.32%. Found: C, 60.40%; H, 4.60%; N, 5.20%.

*N*-Ethyl-2-(6-methoxy-2-oxo-2*H*-chromen-7-yloxy)acetamide (**27**) 

White granular crystals; 30.28% yield; m.p. 171.2–172.3 °C; IR *ν*_max_ (KBr) cm^−1^: 3409, 2933, 1713, 1683, 1591, 1512, 1442, 1421, 1354, 1266, 1243, 1148, 1002, 881. ^1^H-NMR (DMSO-*d*_6_, δ ppm): 1.19 (t, 3H, *J* = 8 Hz, CH_3_), 3.37–3.41 (m, 2H, CH_2_), 3.92 (s, 3H, OCH_3_), 4.53 (s, 2H, CH_2_O), 6.30 (d, 1H, *J* = 12 Hz, C_3_-H), 6.90 (s, 1H, C_8_-H), 7.29 (s, 1H, C_5_-H), 7.63 (d, 1H, *J* = 12 Hz, C_4_-H). ^13^C-NMR (DMSO-*d*_6_, δ ppm): 14.78, 34.05, 56.29, 68.59, 102.59, 108.58, 112.86, 114.50, 143.08, 146.56, 149.39, 150.22, 160.99, 166.94. MS (*m*/*z*): [M]^+^ 277. Anal. Calcd. for C_14_H_15_NO_5_: C, 60.64%; H, 5.45%; N, 5.05%. Found: C, 60.86%; H, 5.45%; N, 4.65%.

2-(6-Methoxy-2-oxo-2*H*-chromen-7-yloxy)-*N*-propylacetamide (**28**)

White granular crystals; 34.42% yield; m.p. 173.2–173.8 °C; IR *ν*_max_ (KBr) cm^−1^: 3422, 3053, 2962, 2876, 1717, 1673, 1611, 1563, 1510, 1427, 1390, 1278, 1251, 1143, 1023, 889. ^1^H-NMR (DMSO-*d*_6_, δ ppm): 0.96 (t, 3H, *J* = 8 Hz, CH_3_), 1.55–1.64 (m, 2H, CH_2_), 3.31–3.36 (m, 2H, CH_2_), 3.95 (s, 3H, OCH_3_), 4.57 (s, 2H, CH_2_O), 6.33 (d, 1H, *J* = 12 Hz, C_3_-H), 6.94 (s, 1H, C_8_-H), 7.31 (s, 1H, C_5_-H), 7.66 (d, 1H, *J* = 12Hz, C_4_-H). ^13^C-NMR (DMSO-*d*_6_, δ ppm): 11.30, 22.75, 40.77, 56.30, 68.63, 102.62, 108.60, 112.87, 114.51, 143.10, 146.58, 149.40, 150.25, 160.99, 167.03. MS (*m*/*z*): [M]^+^ 291. Anal. Calcd. for C_15_H_17_NO_5_: C, 61.85%; H, 5.88%; N, 4.81%. Found: C, 62.05%; H, 5.86%; N, 4.70%.

*N*-Isopropyl-2-(6-methoxy-2-oxo-2*H*-chromen-7-yloxy)acetamide (**29**)

White granular crystals; 19.52% yield; m.p. 197.2–197.5 °C; IR *ν*_max_ (KBr) cm^−1^: 3305, 3077, 2967, 2875, 1722, 1663, 1613, 1558, 1507, 1463, 1422, 1396, 1275, 1248, 1151, 1034, 872. ^1^H-NMR (DMSO-*d*_6_, δ ppm): 1.23 (d, 6H, *J* = 8 Hz, CH_3_), 3.95 (s, 3H, OCH_3_), 4.13–4.25 (m, 1H, CH), 4.55 (s, 2H, CH_2_O), 6.35 (d, 1H, *J* = 8 Hz, C_3_-H), 6.93 (s, 1H, C_8_-H), 7.28 (s, 1H, C_5_-H), 7.65 (d, 1H, *J* = 12Hz, C_4_-H). ^13^C-NMR (DMSO-*d*_6_, δ ppm): 22.68, 41.24, 56.32, 69.00, 103.16, 108.68, 113.02, 114.74, 142.94, 146.69, 149.50, 150.40, 160.94, 166.25. MS (*m*/*z*): [M]^+^ 291. Anal. Calcd. for C_15_H_17_NO_5_: C, 61.85%; H, 5.88%; N, 4.81%. Found: C, 60.90%; H, 5.82%; N, 4.65%.

*N*-Butyl-2-(6-methoxy-2-oxo-2*H*-chromen-7-yloxy)acetamide (**30**)

White powder; 19.59% yield; m.p. 168.4–168.5 °C; IR *ν*_max_ (KBr) cm^−1^: 3426, 3055, 2959, 2934, 2862, 1721, 1675, 1614, 1565, 1511, 1464, 1427, 1391, 1280, 1252, 1146, 1027, 891. ^1^H-NMR (DMSO-*d*_6_, δ ppm): 0.93-0.96 (t, 3H, CH_3_), 1.31–1.42 (m, 2H, CH_2_), 1.51–1.60 (m, 2H, CH_2_), 3.34-3.39 (m, 2H, CH_2_), 3.93 (s, 3H, OCH_3_), 4.56 (s, 2H, CH_2_O), 6.34 (d, 1H, *J* = 8 Hz, C_3_-H), 6.91 (s, 1H, C_8_-H), 7.27 (s, 1H, C_5_-H), 7.63 (d, 1H, *J* = 8Hz, C_4_-H) (Figure S11). ^13^C-NMR (DMSO-*d*_6_, δ ppm): 13.74, 20.01, 31.51, 38.86, 56.32, 69.70, 102.78, 108.63, 112.92, 114.64, 143.00, 146.61, 149.48, 150.28, 160.98, 167.02 (Figure S12). MS (*m*/*z*): [M]^+^ 305. Anal. Calcd. for C_16_H_19_NO_5_: C, 62.95%; H, 6.23%; N, 4.59%. Found: C, 63.15%; H, 6.30%; N, 4.60%.

*N*-Benzyl-2-(6-methoxy-2-oxo-2*H*-chromen-7-yloxy)acetamide (**31**)

White powder; 38.02% yield; m.p. 84.50–86.20 °C; IR *ν*_max_ (KBr) cm^−1^: 3420, 3053, 2931, 2872, 1717, 1671, 1612, 1565, 1508, 1460, 1423, 1389, 1276, 1249, 1143, 1018, 885. ^1^H-NMR (DMSO-*d*_6_, δ ppm): 3.93 (s, 3H, OCH_3_), 4.09 (d, 2H, *J* = 8 Hz, CH_2_), 4.53 (s, 2H, CH_2_O), 6.29 (d, 1H, *J* = 12 Hz, C_3_-H), 6.91 (s, 1H, C_8_-H), 7.28 (s, 1H, C_5_-H), 7.49–7.66 (m, 5H, Ar-H), 8.20 (d, 1H, *J* = 8 Hz, C_4_-H). ^13^C-NMR (DMSO-*d*_6_, δ ppm): 44.58, 56.43, 66.63, 101.66, 108.39, 111.92, 113.73, 124.06, 127.70, 128.91, 131.00, 143.43, 146.72, 149.63, 151.50, 161.46, 167.79. MS (*m*/*z*): [M]^+^ 339. Anal. Calcd. for C_19_H_17_NO_5_: C, 67.25%; H, 5.05%; N, 4.13%. Found: C, 67.28%; H, 4.40%; N, 4.91%.

*N*-(3-Chlorobenzyl)-2-(6-methoxy-2-oxo-2*H*-chromen-7-yloxy)actamide (**32**)

White powder; 11.61% yield; m.p. 155.6–156.0 °C; IR *ν*_max_ (KBr) cm^−1^: 3420, 3053, 2931, 2850, 1716, 1671, 1612, 1567, 1508, 1464, 1423, 1389, 1276, 1248, 1145, 1020, 881. ^1^H-NMR (DMSO-*d*_6_, δ ppm): 3.88 (s, 3H, OCH_3_), 4.44 (d, 2H, *J* = 4 Hz, CH_2_), 4.53 (s, 2H, CH_2_O), 6.28 (d, 1H, *J* = 8 Hz, C_3_-H), 6.87 (s, 1H, C_8_-H), 7.03 (s, 1H, C_5_-H), 7.29–7.40 (m, 3H, Ar-H), 7.46(s, 1H, Ar-H), 7.92 (d, 1H, *J* = 12 Hz, C_4_-H). ^13^C-NMR (DMSO-*d*_6_, δ ppm): 41.79, 56.77, 68.84, 103.20, 107.47, 111.51, 113.42, 126.32, 127.08, 127.68, 130.01, 133.66, 141.07, 143.36, 144.02, 149.70, 150.25, 161.51, 167.30. MS (*m*/*z*): [M]^+^ 373. Anal. Calcd. for C_19_H_16_ClNO_5_: C, 61.13%; H, 4.29%; N, 3.75%. Found: C, 61.87%; H, 4.85%; N, 3.43%.

*N*-(4-Chlorobenzyl)-2-(6-methoxy-2-oxo-2*H*-chromen-7-yloxy)acetamide (**33**)

White sheet-like crystals; 30.12% yield; m.p. 178.3–179.2 °C; IR *ν*_max_ (KBr) cm^−1^: 3420, 3054, 2932, 1716, 1671, 1614, 1566, 1507, 1461, 1423, 1388, 1276, 1248, 1144, 1017, 884. ^1^H-NMR (DMSO-*d*_6_, δ ppm): 3.83 (s, 3H, OCH_3_), 4.53 (d, 2H, *J* = 4Hz, CH_2_), 4.63 (s, 2H, CH_2_O), 6.35 (d, 1H, *J* = 12 Hz, C_3_-H), 6.88 (s, 1H, C_8_-H), 7.16 (s, 1H, C_5_-H), 7.23–7.32 (m, 4H, Ar-H), 7.63 (d, 1H, *J* = 12 Hz, C_4_-H). ^13^C-NMR (DMSO-*d*_6_, δ ppm): 42.42, 56.18, 68.92, 103.16, 108.60, 113.12, 114.84, 128.91, 129.12, 133.60, 136.21, 142.94, 146.60, 149.40, 150.11, 160.91, 167.28. MS (*m*/*z*): [M]^+^ 373. Anal. Calcd. for C_19_H_16_ClNO_5_: C, 61.05%; H, 4.31%; N, 3.75%. Found: C, 61.28%; H, 4.40%; N, 3.59%.

*N*-(3,4-Dichlorobenzyl)-2-(6-methoxy-2-oxo-2*H*-chromen-7-yloxy)acetamide (**34**)

White powder; 36.12% yield; m.p. 161.15–162.25 °C; IR *ν*_max_ (KBr) cm^−1^: 3417, 3040, 2996, 1724, 1678, 1612, 1564, 1507, 1472, 1425, 1389, 1274, 1252, 1145, 1024, 878. ^1^H-NMR (DMSO-*d*_6_, δ ppm): 3.87 (s, 3H, OCH_3_), 4.36 (d, 2H, *J* = 8 Hz, CH_2_), 4.51 (s, 2H, CH_2_O), 6.35 (d, 1H, *J* = 12 Hz, C_3_-H), 6.93 (s, 1H, C_8_-H), 7.30 (s, 1H, C_5_-H), 7.36–7.41 (m, 3H, Ar-H), 7.68 (d, 1H, *J* = 12 Hz, C_4_-H). ^13^C-NMR (DMSO-*d*_6_, δ ppm): 42.18, 56.32, 68.84, 103.04, 108.76, 113.16, 114.68, 127.61, 129.86, 130.63, 131.41, 132.41, 137.78, 143.19, 146.58, 149.33, 150.06, 162.90, 167.47. MS (*m*/*z*): [M − H]^+^ 407. Anal. Calcd. for C_19_H_15_Cl_2_NO_5_: C, 55.90%; H, 3.70%; N, 3.43%. Found: C, 55.50%; H, 3.44%; N, 3.55%.

*N*-(4-Methylbenzyl)-2-(6-methoxy-2-oxo-2*H*-chromen-7-yloxy)acetamide (**35**)

White sheet-like crystals; 65.36% yield; m.p. 171.4–171.6 °C; IR *ν*_max_ (KBr) cm^−1^: 3430, 3055, 2931, 1727, 1677, 1614, 1567, 1507, 1464, 1427, 1389, 1276, 1250, 1146, 1024, 881. ^1^H-NMR (DMSO-*d*_6_, δ ppm): 2.34 (s, 3H, CH_3_), 3.80 (s, 3H, OCH_3_), 5.52 (d, 2H, *J* = 4 Hz, CH_2_), 4.62 (s, 2H, CH_2_O), 6.33 (d, 1H, *J* = 8 Hz, C_3_-H), 6.87 (s, 1H, C_8_-H), 7.15, 7.20 (dd, 4H, *J* = 8 Hz, Ar-H), 7.27 (s, 1H, C_5_-H), 7.63 (d, 1H, *J* = 12 Hz, C_4_-H). ^13^C-NMR (DMSO-*d*_6_, δ ppm): 21.13, 42.88, 56.15, 69.01, 103.18, 108.61, 113.06, 114.73, 127.74, 129.43, 134.62, 137.41, 142.96, 146.67, 149.42, 150.29, 160.92, 167.11. MS (*m*/*z*): [M]^+^ 353. Anal. Calcd. for C_20_H_19_NO_5_: C, 67.98%; H, 5.42%; N, 3.96%. Found: C, 67.69%; H, 5.46%; N, 3.85%.

*N*-(4-Methoxybenzyl)-2-(6-methoxy-2-oxo-2*H*-chromen-7-yloxy)acetamide (**36**)

White sheet crystal; 80.34% yield; m.p. 161.3–161.6 °C; IR *ν*_max_ (KBr) cm^−1^: 3430, 3054, 1727, 1675, 1611, 1567, 1507, 1426, 1388, 1276, 1249, 1144, 1024, 880. ^1^H-NMR (DMSO-*d*_6_, δ ppm): 3.80 (s, 6H, OCH_3_), 4.48 (d, 2H, *J* = 4 Hz, CH_2_), 4.61 (s, 2H, CH_2_O), 6.32 (d, 1H, *J* = 12 Hz, C_3_-H), 6.84–6.88 (m, 2H, Ar-H), 7.11 (s, 1H, C_8_-H), 7.22 (d, 2H, *J* = 8 Hz, Ar-H), 7.28 (s, 1H, C_5_-H), 7.61 (d, 1H, *J* = 8 Hz, C_4_-H). ^13^C-NMR (DMSO-*d*_6_, δ ppm): 42.60, 55.33, 56.16, 69.03, 103.18, 108.63, 113.05, 114.10, 114.68, 129.12, 129.74, 142.95, 146.66, 149.39, 150.29, 159.12, 160.89, 167.07. MS (*m*/*z*): [M]^+^ 369. Anal. Calcd. for C_20_H_19_NO_6_: C, 65.03%; H, 5.19%; N, 3.79%. Found: C, 65.20%; H, 5.24%; N, 3.66%.

*N*-(4-*tert*-Butylbenzyl)-2-(6-methoxy-2-oxo-2*H*-chromen-7-yloxy)acetamide (**37**)

White powder; 50.34% yield; m.p.177.5–178.1 °C; IR *ν*_max_ (KBr) cm^−1^: 3430, 2961, 1730, 1682, 1615, 1568, 1520, 1444, 1428, 1390, 1279, 1249, 1147, 1025, 880. ^1^H-NMR (DMSO-*d*_6_, δ ppm): 1.28 (s, 9H, 3 × CH_3_), 3.79 (s, 3H, OCH_3_), 4.39 (d, 2H, *J* = 8 Hz, CH_2_), 4.61 (s, 2H, CH_2_O), 6.32 (d, 1H, *J* = 8 Hz, C_3_-H), 6.90 (s, 1H, C_8_-H), 7.24 (d, 2H, *J* = 8 Hz, Ar-H), 7.31 (s, 1H, C_5_-H), 7.37 (d, 2H, *J* = 8 Hz, Ar-H), 7.66 (d, 1H, *J* = 12 Hz, C_4_-H). ^13^C-NMR (DMSO-*d*_6_, δ ppm): 31.34, 42.75, 42.88, 56.17, 69.04, 103.15, 108.71, 113.09, 114.62, 125.51, 127.66, 134.40, 143.13, 146.68, 149.38, 150.71, 150.29, 160.98, 167.15. MS (*m*/*z*): [M]^+^ 395. Anal. Calcd. for C_23_H_25_NO_5_: C, 69.86%; H, 6.37%; N, 3.54%. Found: C, 69.91%; H, 6.91%; N, 3.59%.

### 3.3. Acaricidal Activity Assay

*T. cinnabarlnus* was reared on potted young cowpea plants in the laboratory at (26 ± 1) °C and (70 ± 10) % relative humidity (R. H.) and a 14 h:10 h (light:dark) cycle with no acaricide exposure for at least 15 years, which originally collected from field young cowpea plants in Beibei District, Chongqing Municipality, China. 

The slide-dip method [52] was adopted to evaluate the acaricidal activity of **8**–**37** against female adults of *T. cinnabarinus*. The appropriate amounts of target compounds were dissolved in 0.2 mL acetone and then diluted with water containing 0.1% Tween-80 to obtain the desired final concentration of 1000 mg/L for the preliminary screening. Based on the preliminary test results, a series of five to seven concentrations of the tested compounds were chosen to determine the median lethal concentration (LC_50_) values of the compounds. Propargite 90.00% TC and scopoletin were used as positive controls, and water containing 0.1% Tween-80 was used as a blank control. Acaricidal activity assays were performed in triplicate and repeated thrice. The LC_50_ values of the tested compounds were calculated using the probit analysis procedure of SPSS 17.0 for Windows (SPSS Inc., Chicago, IL, USA).

The leaf-dip method was used to evaluate the acaricidal activity of compound **8** against eggs, larval, and nymphal of *T. cinnabarinus*. The test solutions of compound **8** was prepared as above slide-dip method. Leaf discs were prepared to obtain uniform individuals at different developmental stages. Fresh cowpea leaves that had not been exposed to pesticides were washed thoroughly. Leaf discs with 3 cm diameters were placed on a corresponding size water-saturated sponge in a Petri dish (9 cm diameter) [53]. Adult females (20–30) were transferred to each leaf disc, allowed to lay eggs, and removed after 12 h. The leaf disc with eggs, larvae, and nymphs were then dipped in the compound **8** solutions for 5 s, taken out, and then laid on sponge in Petri dish again. The observed results were recorded after 48 h.

### 3.4. 2D- and 3D-QSAR Study

#### 3.4.1. Data Set

The synthesized thirty target compounds and their acaricidal activities (LC_50_ values) were used as data set for QSAR analysis. They were randomly divided into a 25-molecule training set for 2D- and 3D-QSAR models development and 5-molecule test set (compounds **11**, **16**, **19**, **27** and **33**) for external validation.

#### 3.4.2. 2D-QSAR (Multiple Linear Regression Model) Method

2D structures of the 30 target synthesized compounds were generated by ChemDraw Ultra (Cambridge Soft Corporation, Cambridge, MA, USA), and their energies were minimized using MM2 of Chem3D Ultra. Then 1666 molecular descriptors were calculated for each compound using DRAGON Web version 1.0 developed by the Milano Chemometrics and QSAR Research Group (http://www.vcclab.org/lab/edragon/start.html). These descriptors included (i) 0D constitutional (atom and group counts), (ii) 1D functional groups and atom-centred fragments, (iii) 2D topological, counts, autocorrelations, connectivity indices, information indices, topological indices, and eigenvalue-based indices, and (iv) 3D geometrical, WHIM, and GETAWAY descriptors, etc. [54]. 1302 descriptors were utilized as input values for model construction after eliminating the descriptors with constant values or mostly zero values (>90%) from the all the calculated descriptors.

2D-QSAR models were obtained using SPSS software (Version 17.0) that can run multiple linear regression. Different mathematical transformations of the observed median lethal concentration (LC_50_) of the training set analogs, including property LC_50_ (mg/L), LC_50_ (mol/L), 1/LC_50_ (mg/L), 1/LC_50_ (mol/L), log LC_50_ (mg/L), log LC_50_ (mol/L), −log LC_50_ (mg/L) and −log LC_50_ (mol/L) values, were utilized in the present 2D-QSAR modeling to searching for the best model. −log LC_50_ (mol/L) (pLC_50_) values were used as dependent variables. Stepwise method for variable selection along with multiple linear regression was used to construct models.

#### 3.4.3. 3D-QSAR (CoMFA and CoMSIA) Methods

The molecular structures of synthesized compounds were generated and optimized using SYBYL 6.9 (Tripos Associates, St. Louis, MO, USA). The Gasteiger–Hückel charge, Tripos force field, and Powell method were used for structure optimization. To guarantee the obtaining of the molecular lowest energy conformation, conformation search was executed by using multisearch routin [55]. The most important component of a 3D-QSAR study is the alignment of the molecules based on the scaffold they share [56]. In this paper, the 7-oxy-6-methoxy-2*H*-chromen-2-one structure was selected as the common scaffold for molecular alignment. Compound **8** was used as the template molecule. All other synthesized acaricidal agents were aligned with the 7-oxy-6-methoxy-2*H*-chromen-2-one core. 

The comparative molecular field analysis (CoMFA) and comparative molecular similarity indices analysis (CoMSIA) are commonly used 3D-QSAR methods [51]. In CoMFA, the steric and electrostatic fields were calculated by setting the energy cutoff as the default value of 30 kcal·mol^−1^. Five CoMSIA fields including the steric, electrostatic, hydrophobic, hydrogen-bond donor and hydrogen-bond acceptor were calculated using the default attenuation factor of 0.3 for Gaussian function. Field type “Stdev * Coeff” was used as the coefficient to analysis the contour map of each field [36]. The partial least squares (PLS) [57] was used to quantify the relationships by setting the biological activity (pLC50 values) as the dependent variables and the CoMFA/CoMSIA descriptors as independent variables.

### 3.5. Molecular Docking

Molecular docking studies were performed using AutoDock 4.2 and AutoDock Tools version 1.5.6 (ADT). The 3D structure of *TcPMCA1* (GenBank No. KP455490), and its binding pocket were obtained from the I-TASSER server (Available online: http://zhanglab.ccmb.med.umich.edu/I-TASSER/), then water molecules were removed, polar hydrogen atoms were added, Compute Gasteiger charges were added, and AD 4 type atoms were assigned [41]. The 3D structure of ligands were constructed and their energy minimization were performed using ChemOffice 2004. Following by the structural optimization, all ligands were prepared for docking by merging non-polar hydrogen atoms, detecting rotatable bonds and adding gasteiger charges [41]. The grid box size of 60 × 60 ×60 Å was generated and allocated to center of binding cavity using x, y and z coordinates of 102.273, 100.115, and 118.080 for intend searching modality. Other parameters were set as the default. The Lamarckian genetic algorithmwas applied to calculate the possible conformation of the ligand molecule and macromolecule. Finally, the docking results were analyzed using the free version of Discovery Studio Visualizer 4.5 (Accelrys Software Inc., San Diego, CA, USA) [58].

### 3.6. In Silico ADME Prediction

On the basis of Lipinski’s rule of five and its extensions [59], we calculated molecular volume (MV), molecular weight (MW), logarithm of partition coefficient (miLogP), number of hydrogen bond acceptors (n-ON), number of hydrogen bonds donors (n-OHNH), topological polar surface area (TPSA), number of rotatable bonds (n-ROTB) and Lipinski’s rule of five using Molinspiration online property calculation toolkit [60]. Absorption (% ABS) was calculated as follows: % ABS = 109 − (0.345 × TPSA) [61].

## 4. Conclusions

Thirty phenolic ether derivatives of scopoletin including twelve compounds with amide groups were synthesized successfully using a molecular hybridization method. Their acaricidal activities, QSAR, molecular docking and a silico ADME properties were investigated. Some of these compounds exhibit more pronounced acaricidal activity than scopoletin, especially compounds **32, 20**, **28**, **27** and **8** exhibited about 8.41-, 7.32-, 7.23-, 6.76-, and 6.65-fold higher acaricidal potency than scopoletin. Compound **32** possessed the the most promising acaricidal activity and exhibited about 1.45-fold higher acaricidal potency against *T. cinnabarinus* than propargite. Statistically significant 2D-QSAR model supports the observed acaricidal activities and reveals that polarizability (HATS5p) was the most important parameter controlling bioactivity. 3D-QSAR (CoMFA: q^2^ = 0.802, r^2^ = 0.993; CoMSIA: q^2^ = 0.735, r^2^ = 0.965) results show that bulky substituents at R_4_, R_1_, R_2_ and R_5_ (C_6_, C_3_, C_4_, and C_7_) positions, electron positive groups at the R_5_ (C_7_) position, hydrophobic groups at the R_1_ (C_3_) and R_2_ (C_4_), H-bond donors groups at R_1_ (C_3_) and R_4_ (C_6_) will increase their acaricidal activity, which provide a good insight into the molecular features relevant to the acaricidal activity for further designing novel acaricidal agents. Molecular docking demonstrates that these selected derivatives display different bide modes with *TcPMCA1* from lead compound and they interact with more key amino acid residues than scopoletin. In silico ADME properties study of scopoletin and its phenolic ether derivatives were also analyzed and showed potential to develop these compounds as good acaricidal candidates.

## Figures and Tables

**Table 1:** Contact activity of scopoletin and its phenolic ether derivatives (**8**–**37**) against female adults of *T. cinnabarinus* (48 h).

Compounds	R	LC_50_ (mmol/L)	pLC_50_ (mol/L)	χ^2^	*P*
**8**	methyl	0.829 ± 0.169	3.0813	1.678	0.642
**9**	ethyl	0.840 ± 0.184	3.0757	4.958	0.175
**10**	*n*-propyl	2.326 ± 0.522	2.6333	2.125	0.547
**11**	isopropyl	1.767 ± 0.254	2.7527	1.187	0.756
**12**	*n*-butyl	1.253 ± 0.292	2.9022	4.875	0.181
**13**	isobutyl	3.021 ± 0.410	2.5199	1.633	0.652
**14**	methyl cyclopropane	2.875 ± 0.579	2.5414	1.929	0.587
**15**	cyclopentyl	6.588 ± 0.929	2.1812	0.114	0.990
**16**	cyclohexyl	2.464 ± 0.549	2.6083	1.008	0.799
**17**	methyl cyclohexane	3.586 ± 0.743	2.4454	2.495	0.476
**18**	benzyl	2.557 ± 0.363	2.5922	3.133	0.372
**19**	4-CH_3_- benzyl	3.196 ± 0.808	2.4954	3.418	0.332
**20**	4-C(CH_3_)-benzyl	0.753 ± 0.105	3.1229	3.475	0.324
**21**	4-NO_2_-benzyl	1.686 ± 0.412	2.7730	1.929	0.587
**22**	3,4-2Cl-benzyl	1.652 ± 0.222	2.7821	2.889	0.409
**23**	4-Cl-benzyl	4.208 ± 0.872	2.3759	0.497	0.919
**24**	4-CF_3_-benzyl	1.139 ± 0.218	2.9435	5.571	0.134
**25**	4-OCF_3_-benzyl	2.853 ± 0.560	2.5447	0.567	0.904
**26**	methyl	2.541 ± 0.467	2.5950	3.990	0.263
**27**	ethyl	0.815 ± 0.230	3.0888	5.424	0.143
**28**	*n*-propyl	0.762 ± 0.0937	3.1180	3.764	0.288
**29**	isopropyl	3.045 ± 0.799	2.5165	2.416	0.491
**30**	*n*-butyl	1.015 ± 0.262	2.9935	1.689	0.639
**31**	benzyl	1.134 ± 0.1149	2.9452	2.428	0.297
**32**	3-Cl-benzyl	0.655 ± 0.2539	3.1835	2.280	0.516
**33**	4-Cl-benzyl	0.848 ± 0.1359	3.0716	2.642	0.45
**34**	3,4-2Cl-benzyl	0.963 ± 0.2437	3. 0165	1.208	0.598
**35**	4-CH_3_-benzyl	1.774 ± 0.3783	2.7510	1.330	0.722
**36**	4-OCH_3_-benzyl	1.516 ± 0.2618	2.8193	0.062	0.996
**37**	4-C(CH_3_)-benzyl	3.601 ± 0.4786	2.4436	3.129	0.209
Scopoletin	—	5.510 ± 1.553	2.2588	0.416	0.937
Propargite	—	0.953 ± 0.1093	3.0209	4.034	0.258

**Table 2:** Acaricidal activity of compound **8** against eggs, larvae, and nymphs of *T. cinnabarinus* (48 h).

Stages of *T. cinnabarinus*	LC_50_ (mmol/L)	pLC_50_ (mol/L)	χ^2^	*P*
Eggs	Non-ovicidal	—	—	—
Larva	0.679 ± 0.2066	3.1682	3.268	0.195
Nymph	3.889 ± 1.059	2.4101	5.177	0.075

**Table 3:** Selected descriptors of multiple linear regression.

Descriptor	Chemical Meaning	Type	Sig.	t	VIF
constant	Intercept		0.000	6.026	
R8e	R autocorrelation of lag 8/weighted by Sanderson electronegativity	GETAWAY descriptor	0.000	−4.787	1.314
HATS5p	Leverage-weighted autocorrelation of lag5/weighted by polarizability	GETAWAY descriptor	0.000	6.725	1.479
Depressant-80	Ghose-Viswanadhan-Wendoloski antidepressant-like index at 80%	Drug-like indices	0.000	5.061	1.605
MATS6e	Moran autocorrelation of lag 6 weighted by Sanderson electonegativity	2D autocorrelations	0.002	−3.514	1.032
HNar	Narumi harmonic topological index	Topological indices	0.005	−3.178	1.439

**Table 4:** The correlation matrix of descriptors.

	R8e	HATS5p	Depressant-80	MATS6e	HNar
R8e	1	−0.314	0.165	−0.023	0.391
HATS5p	−0.314	1	−0.494	−0.080	−0.141
Depressant-80	0.165	−0.494	1	0.099	0.398
MATS6e	−0.023	−0.080	0.099	1	−0.092
HNar	0.391	−0.141	0.398	−0.092	1

**Table 5:** Values of significant molecular descriptors, experimental and predicted acaricidal activities (pLC_50_, mol/L) from 2D-QSAR of the compounds **8**–**37**.

Compounds	R8e	HATS-5p	Depressant-80	MATS-6e	HNar	Experimental Activity	Prediced Activity	Residual
**8**	0.384	0.118	0	−0.02	1.837	3.0813	3.0281	0.0532
**9**	0.706	0.146	0	−0.03	1.846	3.0757	3.0818	−0.0061
**10**	0.749	0.116	0	−0.043	1.855	2.6333	2.6575	−0.0242
**11**	0.919	0.117	0	−0.043	1.789	2.7527	2.5732	0.1795
**12**	0.81	0.117	1	0.004	1.862	2.9022	2.8258	0.0764
**13**	0.821	0.075	1	−0.055	1.8	2.5199	2.3743	0.1456
**14**	0.807	0.098	1	−0.055	2	2.5414	2.4945	0.0469
**15**	0.955	0.099	1	−0.066	2	2.1812	2.4751	−0.2939
**16**	0.99	0.097	1	−0.034	2	2.6083	2.2726	0.3357
**17**	0.83	0.103	1	0.025	2	2.4454	2.4343	0.0111
**18**	0.747	0.097	1	0.025	2	2.5922	2.4056	0.1866
**19**	0.678	0.1	1	−0.017	1.941	2.4954	2.6896	−0.1942
**20**	0.66	0.112	1	0.019	1.84	3.1229	2.9197	0.2032
**21**	0.695	0.102	1	−0.013	1.895	2.7730	2.7490	0.024
**22**	0.755	0.117	1	0.098	1.89	2.7821	2.7227	0.0594
**23**	0.747	0.098	1	0.145	1.941	2.3759	2.3697	0.0062
**24**	0.674	0.097	1	−0.115	1.84	2.9435	2.9037	0.0398
**25**	0.74	0.114	0	0.001	1.846	2.5447	2.5984	−0.0537
**26**	0.761	0.118	0	0.05	1.81	2.5950	2.5985	−0.0035
**27**	0.668	0.117	1	0.08	1.818	3.0888	2.9454	0.1434
**28**	0.609	0.115	1	0.037	1.826	3.1180	3.0118	0.1062
**29**	0.59	0.092	1	0.106	1.775	2.5165	2.8367	−0.3202
**30**	0.604	0.109	1	0.015	1.833	2.9935	2.9747	0.0188
**31**	0.586	0.108	1	−0.01	1.948	2.9452	2.8549	0.0903
**32**	0.567	0.133	1	−0.063	1.902	3.1835	3.4251	−0.2416
**33**	0.589	0.113	1	−0.004	1.902	3.0716	2.9827	0.0889
**34**	0.591	0.124	0	−0.052	1.862	3.0165	2.9033	0.1132
**35**	0.568	0.103	1	−0.012	1.902	2.7510	2.9068	−0.1558
**36**	0.625	0.113	1	0.019	1.906	2.8193	2.9159	−0.0966
**37**	0.693	0.105	0	0.045	1.822	2.4436	2.5076	−0.064

**Table 6:** Summary of the results obtained from CoMFA and CoMSIA analyses.

Parameter	CoMFA	CoMSIA
R^2^ (correlation coefficient squared)	0.993	0.965
ONC (the optimal number of components)	6	6
q^2^_LOO_ (leave-one-out cross validation correlation coefficient squared)	0.802	0.735
F value	422.047	83.553
SEE	0.029	0.059
Fields	SE	SEHDA
Field distribution (%)		
Steric	70.8	21.5
Electrostatic	29.2	28.5
Hydrophobic	–	44.9
Donor	–	0
Acceptor	–	5.0
Testing set		
R^2^_pred_	0.999	0.787

**Table 7:** Experimental and predicted acaricidal activities (pLC_50_, mol/L) from 3D-QSAR of the compounds **8**–**37**.

Compounds	Experimental pLC_50_	CoMFA		CoMSIA	
		Prediced pLC_50_	Residual	Prediced pLC_50_	Residual
**8**	3.0813	3.0973	−0.016	3.0924	−0.0111
**9**	3.0757	3.0725	0.0032	3.0575	0.0182
**10**	2.6333	2.6274	0.0059	2.5286	0.1047
**11**	2.7527	2.7598	−0.0071	2.7593	−0.0066
**12**	2.9022	2.9078	−0.0056	2.9684	−0.0662
**13**	2.5199	2.4480	0.0719	2.4733	0.0466
**14**	2.5414	2.5493	−0.0079	2.5388	0.0026
**15**	2.1812	2.2194	−0.0382	2.2796	−0.0984
**16**	2.6083	2.6112	−0.0029	2.8778	−0.2695
**17**	2.4454	2.4623	−0.0169	2.4475	−0.0021
**18**	2.5922	2.6016	−0.0094	2.5501	0.0421
**19**	2.4954	2.4951	0.0003	2.5121	−0.0167
**20**	3.1229	3.1170	0.0059	3.1055	0.0174
**21**	2.773	2.7801	−0.0071	2.7715	0.0015
**22**	2.7821	2.7970	−0.0149	2.8082	−0.0261
**23**	2.3759	2.3723	0.0036	2.3906	−0.0147
**24**	2.9435	2.9283	0.0152	2.9396	0.0039
**25**	2.5447	2.5653	−0.0206	2.5621	−0.0174
**26**	2.595	2.5766	0.0184	2.5922	0.0029
**27**	3.0888	3.0794	0.0094	3.0367	0.0521
**28**	3.118	3.1368	−0.0188	3.0989	0.0191
**29**	2.5165	2.5110	0.0055	2.5584	−0.0419
**30**	2.9935	2.9990	−0.0055	2.9795	0.0140
**31**	2.9452	2.9766	−0.0314	2.9259	0.0193
**32**	3.1835	3.1459	0.0376	3.0742	0.1093
**33**	3.0716	3.0717	−0.00005	3.1080	−0.0364
**34**	3.0165	3.0186	−0.0021	3.0850	−0.0685
**35**	2.751	2.7630	−0.012	2.8301	−0.0791
**36**	2.8193	2.7858	0.0335	2.8629	−0.0436
**37**	2.4436	2.4378	0.0058	2.3760	0.0676

**Table 8:** Binding energy and nonbonding interaction of scopoletin and its derivatives.

Compounds	Binding Energy	Van der Waals	H-Bond		Hydrophobic Interaction (Pi–Alkyl and Alkyl)		Electrostatic Interaction (Pi–Anion and Pi–Cation)	
			Amino Acid-Ligand Atom	Distance (Å)	Amino Acid-Ligand Atom	Distance (Å)	Amino Acid-Ligand Atom	Distance (Å)
**4**	−5.25	GLU214, SER215, HIS223, SER221, ARG781	ASP222 [C-O…H] ASP213 [C-O…H] * GLU220 [C-O…H] *	2.05 2.81 3.33	VAL224 [Alkyl–Pi] THR218 [Sigma–Pi]	4.07, 4.88 2.23		
**8**	−5.15	SER215, HIS223, THR218, ARG781, ASN725, GLU220, SER221	GLU214 [N-H…O] ASP222 [N-H…O] ASP213 [C-H…O] * GLY723 [C-O…H] * ASP724 [C-O…H] *	2.15 1.97 2.75 3.31 3.11	VAL224 [Alkyl–Pi]	4.53, 5.47		
**9**	−5.38	SER215, THR218, ASN725, MET490, GLU220, SER 221, HIS223	GLU214 [N-H…O] ASP222 [N-H…O] ASP213 [C-H…O] * GLY723 [C-O…H] * ASP724 [C-O…H] *	2.12 1.97 2.76 3.66 3.25	VAL224 [Alkyl–Pi]	4.50, 5.39		
**12**	−5.40	SER215, HIS223, THR218, ASN725, GLY723, LYS481, LYS549, GLU220, SER221	GLU214 [N-H…O] ASP222 [N-H…O] ASP213 [C-H…O] *ASP724 [C-O…H] *	2.03 2.03 2.55 3.19	VAL224 [Alkyl–Pi] MET490 [Alkyl–Alkyl]	4.17, 5.20 5.08		
**20**	−7.41	SER215, SER221, GLU220, MET490, ILE732, ASP698, THR728, GLU697, GLY648, LEU649, ARG781, ASN725, THR218, HIS223	GLU214 [N-H…O] ASP222 [N-H…O] ASP213 [C-H…O] * GLY723 [C-O…H] *	1.98 2.05 2.48 3.16	VAL224 [Alkyl–Pi]	4.27, 5.47	LYS481[Cation–Pi] ASP724[Anion–Pi]	4.54 4.45
**28**	−6.22	ILE212, HIS223, ASP222, GLU220, ASN725, ASP724, PRO784, SER783, SER782	GLU214 [N-H…O] VAL224 [N-H…O] THR218 [C-O…H] ASP213 [C-H…O] * GLY723 [C-O…H] * ARG781 [C-H…O] * GLU214 [C-O…H] *	1.77 2.96 2.15 2.53 3.16 2.21 3.53	VAL224 [Alkyl–Pi]LEU217 [Alkyl–Alkyl]	3.78, 4.45 4.78		

* Nonconventional hydrogen bond.

**Table 9:** Evaluation parameters of Lipinski’s rule of five and its extensions from scopoletin and its phenolic ether derivatives (**8**–**37**).

Entry	miLogp	%ABS	TPSA (A^2^)	MW	n-ON Acceptors	nOHNH Donors	Lipinski’s Violations	n-ROTB	MV
Rule	≤5	—	≤140	≤500	≤10	≤5	≤1	≤10	—
**4**	1.33	88.41	59.67	192.17	4	1	0	1	162.15
**8**	1.64	92.21	48.68	206.20	4	0	0	2	179.68
**9**	2.01	92.21	48.68	220.22	4	0	0	3	196.48
**10**	2.52	92.21	48.68	234.25	4	0	0	4	213.28
**11**	2.38	92.21	48.68	234.25	4	0	0	3	213.07
**12**	3.07	92.21	48.68	248.28	4	0	0	5	230.08
**13**	2.76	92.21	48.68	248.28	4	0	0	4	229.87
**14**	2.51	92.21	48.68	246.26	4	0	0	4	219.51
**15**	3.04	92.21	48.68	260.29	4	0	0	3	236.31
**16**	3.54	92.21	48.68	274.32	4	0	0	3	253.11
**17**	3.92	92.21	48.68	288.34	4	0	0	4	269.91
**18**	3.23	92.21	48.68	282.30	4	0	0	4	251.33
**19**	3.68	92.21	48.68	296.32	4	0	0	4	267.89
**20**	4.94	92.21	48.68	338.40	4	0	0	5	317.51
**21**	3.19	76.40	94.50	327.29	7	0	0	5	274.66
**22**	4.51	92.21	48.68	351.19	4	0	0	4	278.40
**23**	3.91	92.21	48.68	316.74	4	0	0	4	264.86
**24**	4.13	92.21	48.68	350.29	4	0	0	5	282.62
**25**	4.20	89.02	57.91	366.29	5	0	0	6	291.61
**26**	0.74	82.17	77.78	263.25	6	1	0	4	227.87
**27**	1.12	82.17	77.78	277.28	6	1	0	5	244.67
**28**	1.62	82.17	77.78	291.30	6	1	0	6	261.47
**29**	1.42	82.17	77.78	291.30	6	1	0	5	261.25
**30**	2.18	82.17	77.78	305.33	6	1	0	7	278.27
**31**	2.14	82.17	77.78	339.35	6	1	0	6	299.51
**32**	2.79	82.17	77.78	373.79	6	1	0	6	313.05
**33**	2.82	82.17	77.78	373.79	6	1	0	6	313.05
**34**	3.42	82.17	77.78	408.24	6	1	0	6	326.59
**35**	2.59	82.17	77.78	353.37	6	1	0	6	316.08
**36**	2.20	78.98	87.01	369.37	7	1	0	7	325.06
**37**	3.85	82.17	77.78	395.45	6	1	0	7	365.70

Note: % ABS: percentage absorption, TPSA: topological polar surface area, n-ROTB: number of rotatable bonds, MV: molecular volume, MW: molecular weight, milogP: logarithm of partition coefficient of compound between n-octanol and water, n-ON acceptors: number of hydrogen bond acceptors, n-OHNH donors: number of hydrogen bonds donors.

## Supplementary Materials

^1^H-NMR and ^13^C-NMR of representive compounds.
