# Supplementary material for: Synthesis and Acaricidal Activities of Scopoletin Phenolic Ether Derivatives: QSAR, Molecular Docking Study and in Silico ADME Predictions
Source: Molecules. 2018 Apr 24;23(5):995. doi: 10.3390/molecules23050995 (PMC6102537; doi:10.3390/molecules23050995)
Supplement: Supplementary file 1 [file molecules-23-00995-s001.pdf]

Supplementary Data

Synthesis and Acaricidal Activities of Scopoletin Phenolic Ether Derivatives: QSAR, Molecular Docking Study and in Silico ADME Predictions

Jinxiang Luo †, Ting Lai †, Tao Guo, Fei Chen, Linli Zhang, Wei Ding and Yongqiang Zhang *

College of Plant Protection, Southwest University, Chongqing 400715, China; xiangxiangnx@163.com (J.L.); laiting93@163.com (T. L.); 13994888326.guotao@163.com (T.G.); cf759974605@126.com (F.C.); zhll87_9@163.com (L. Z.); dwing818@163.com (W.D.)

* Corresponding author: zyqiang@swu.edu.cn; Tel./Fax: +86 023 6825 0218

† These two authors contributed equally to this work

Received: 3 April 2018; Accepted: 18 April 2018; Published: date

^{1}H-NMR and ^{13}C-NMR of representative compounds

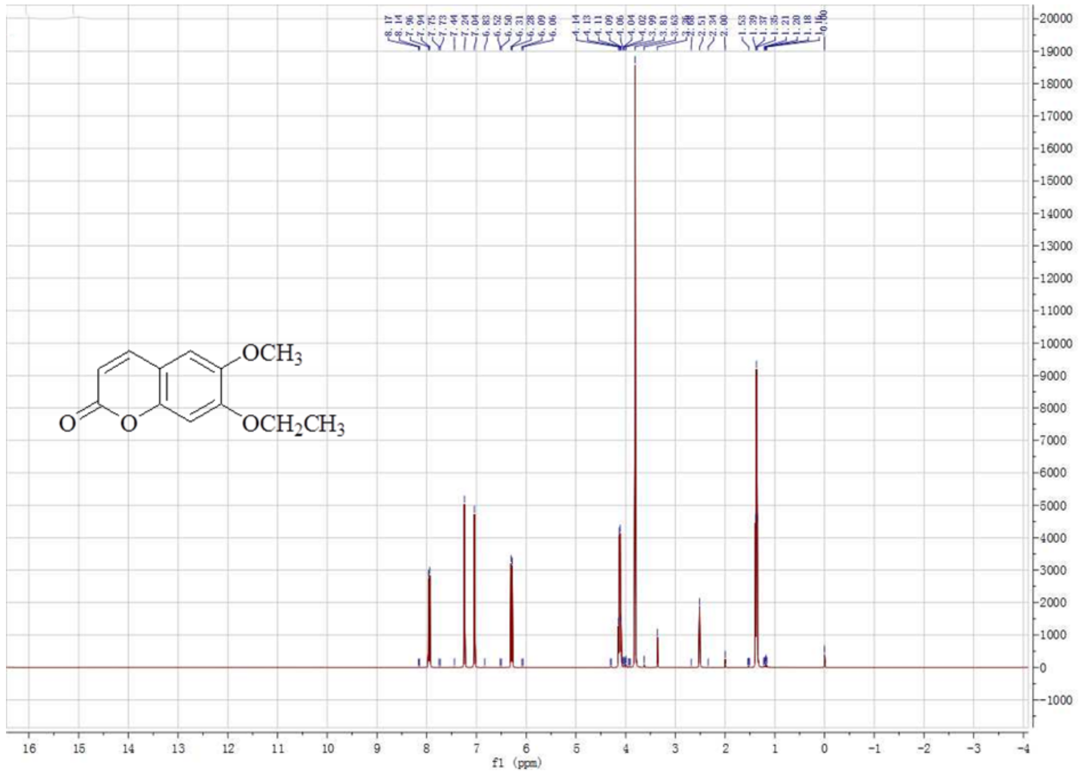

Figure S1. ^{1}H-NMR of 7-ethoxy-6-methoxy-2*H*-chromen-2-one (**9**)

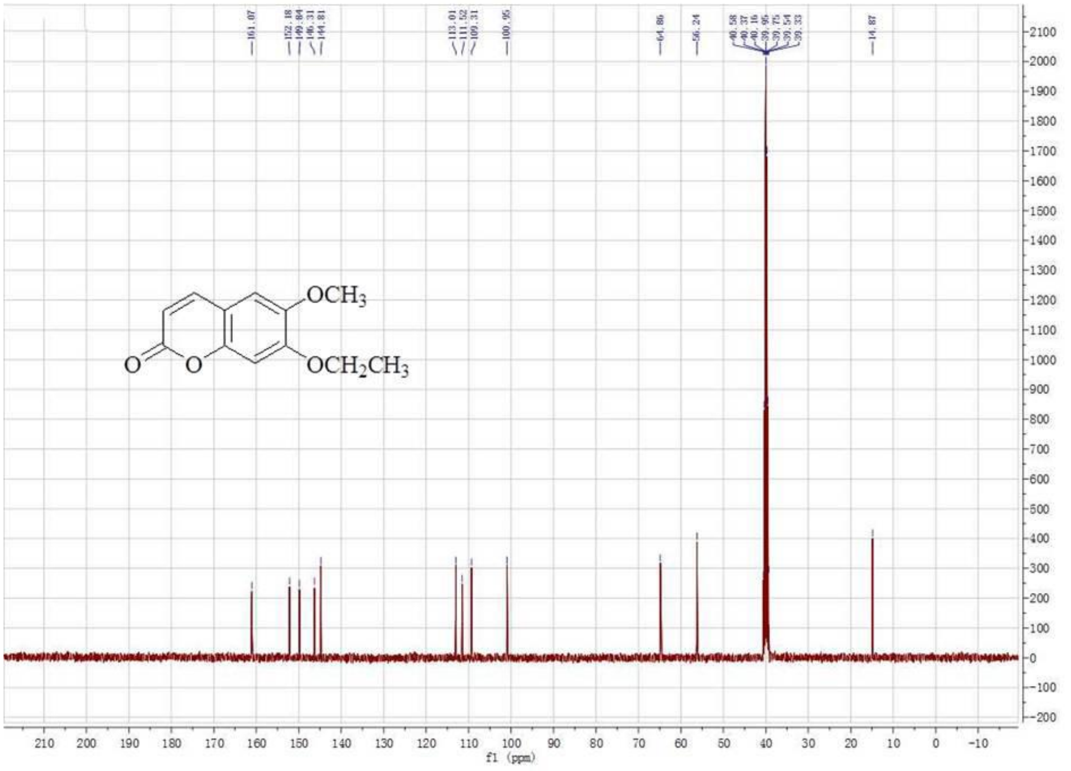

Figure S2. ^{13}C-NMR of 7-ethoxy-6-methoxy-2*H*-chromen-2-one (**9**)

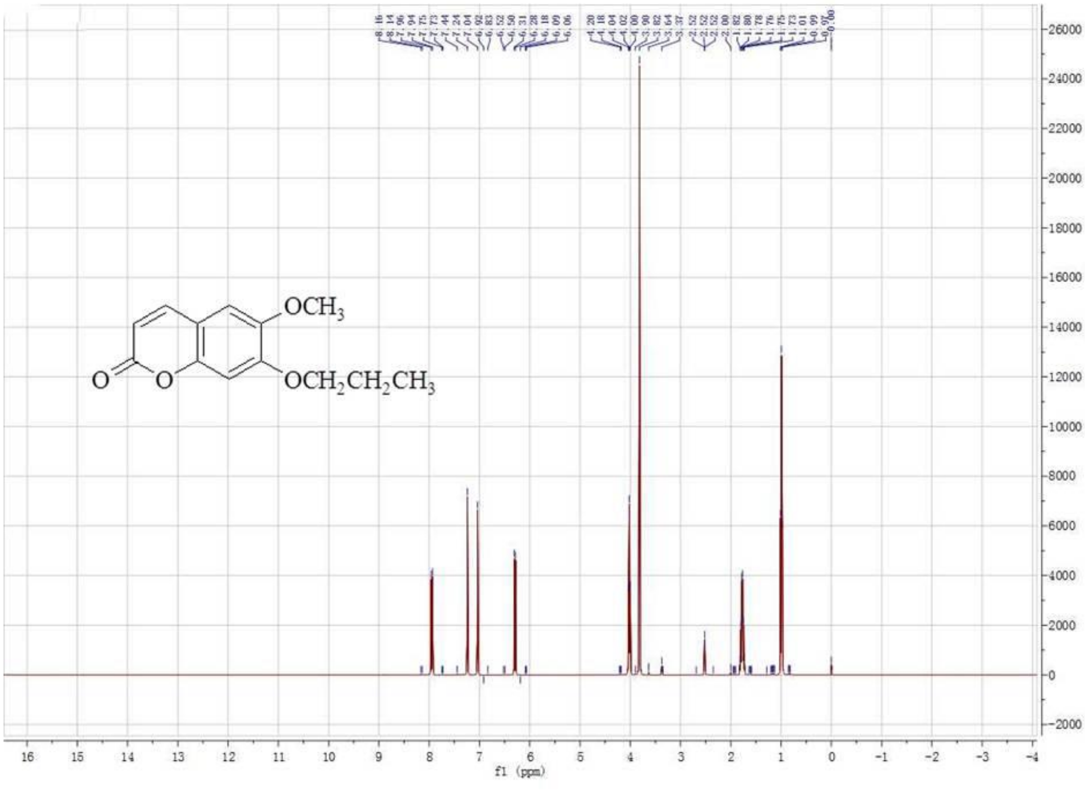

Figure S3. ^{1}H-NMR of 6-methoxy-7-propoxy-2*H*-chromen-2-one (**10**)

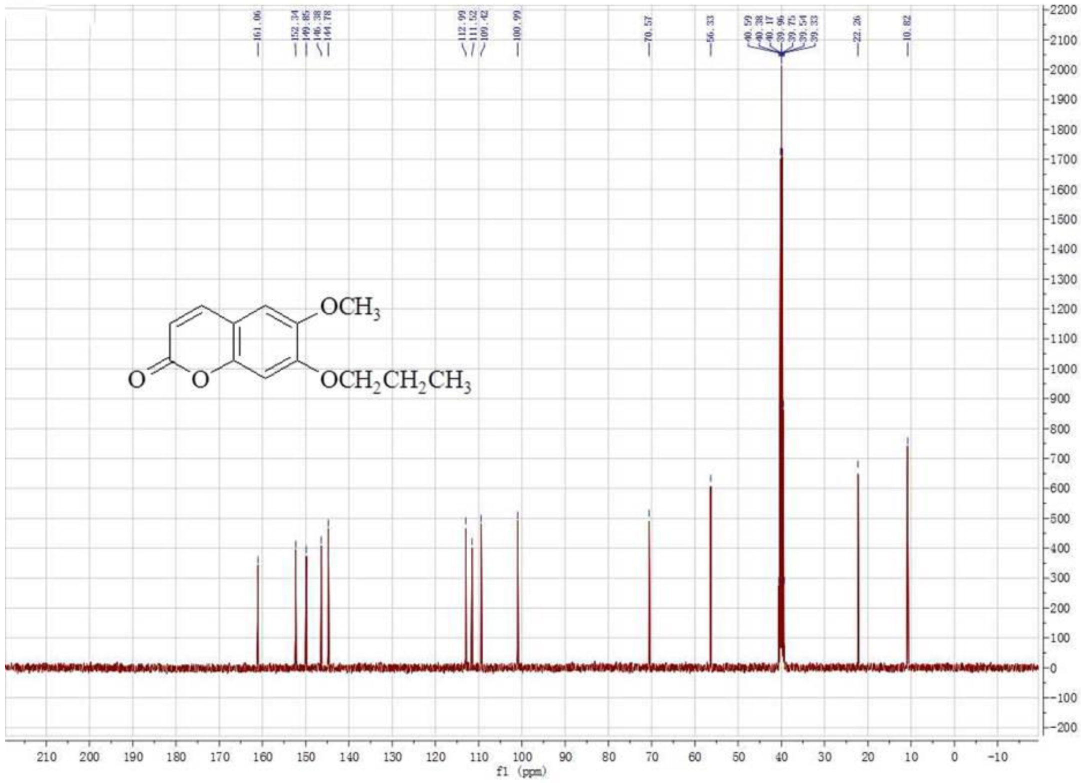

Figure S4. ^{13}C-NMR of 6-methoxy-7-propoxy-2*H*-chromen-2-one (**10**)

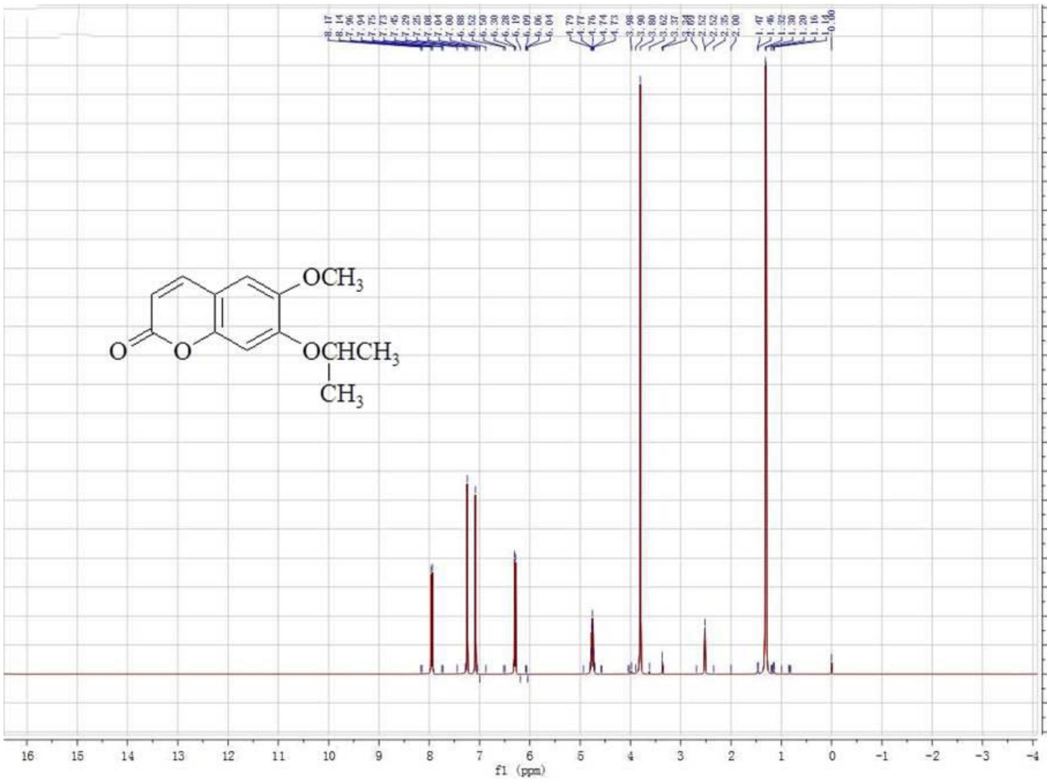

Figure S5. ^{1}H-NMR of 7-Isopropoxy-6-methoxy-2*H*-chromen-2-one (**11**)

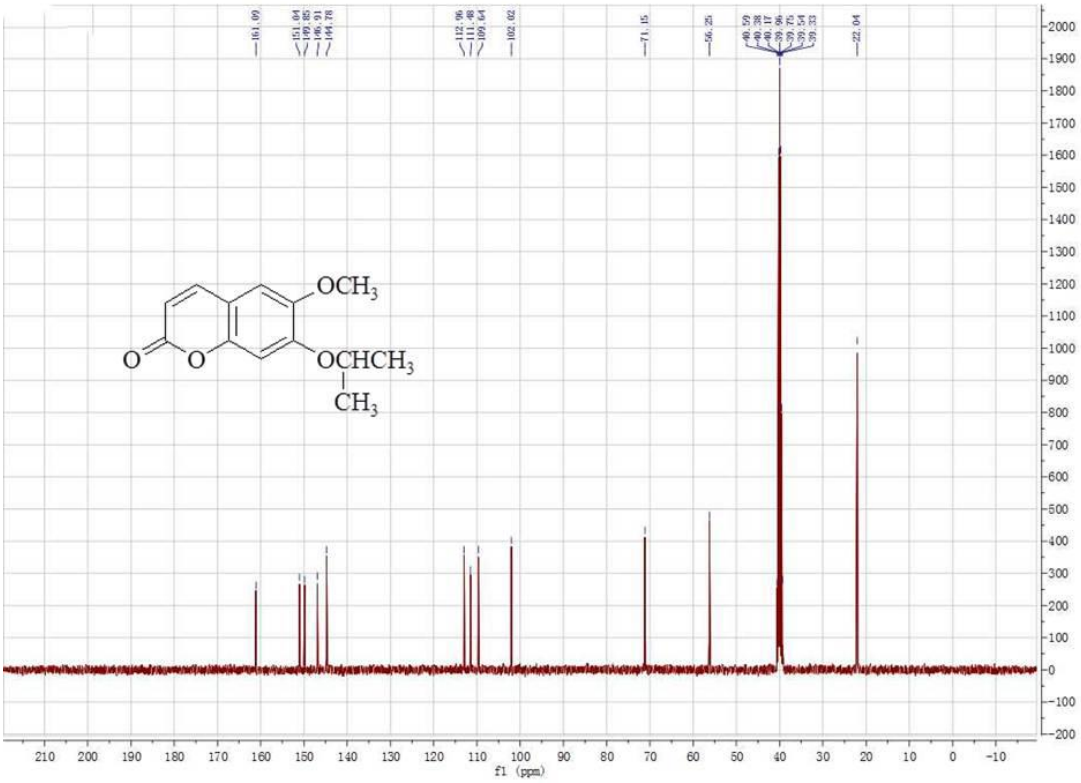

Figure S6.^{13}C-NMR of 7-isopropoxy-6-methoxy-2*H*-chromen-2-one (**11**)

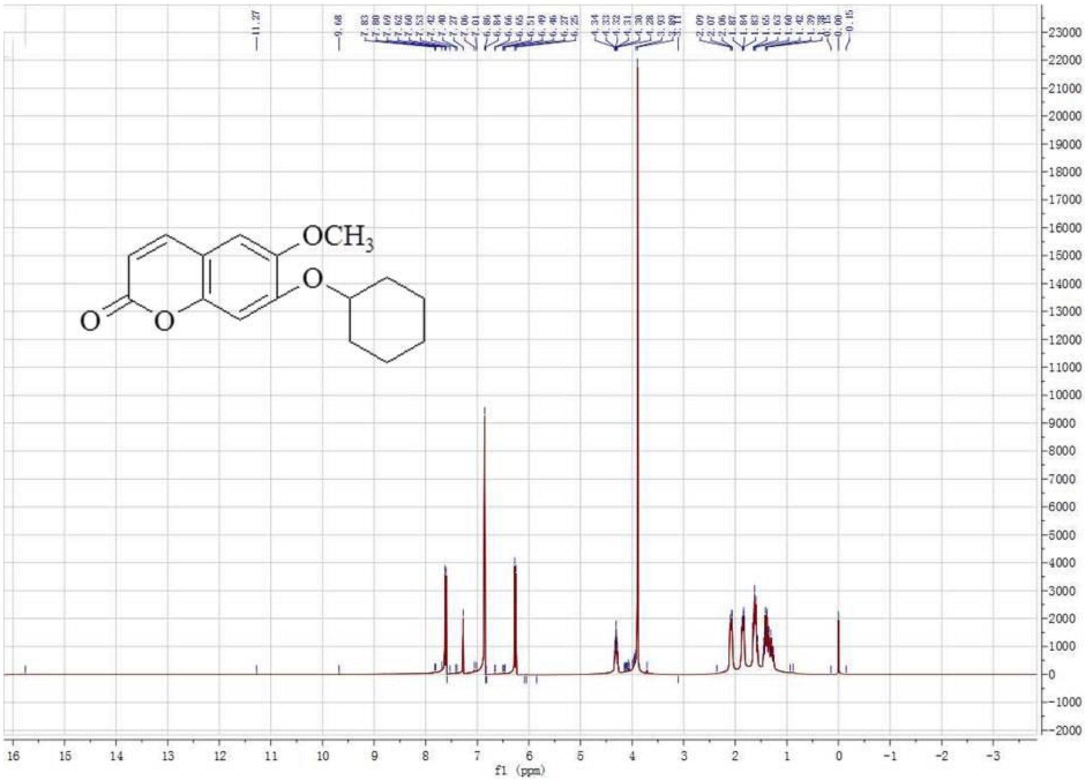

Figure S7. ^{1}H-NMR of 7-(cyclohexyloxy)-6-methoxy-2*H*-chromen-2-one (**16**)

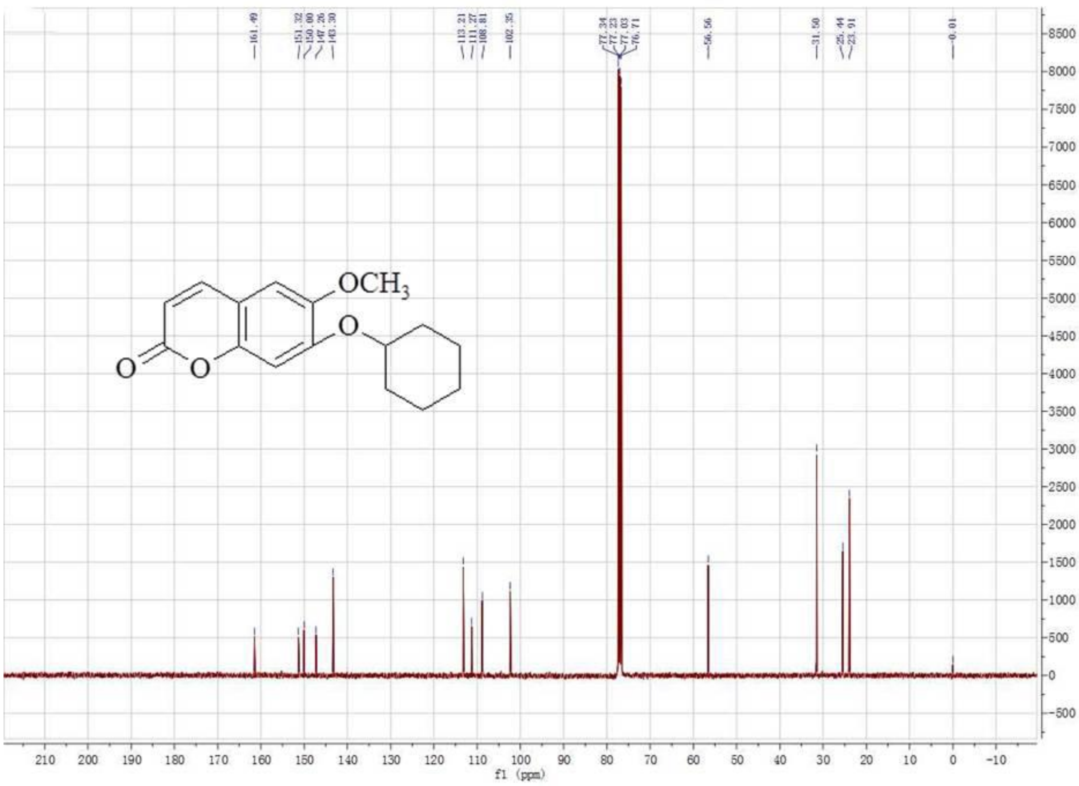

Figure S8.^{13}C-NMR of 7-(cyclohexyloxy)-6-methoxy-2*H*-chromen-2-one (**16**)

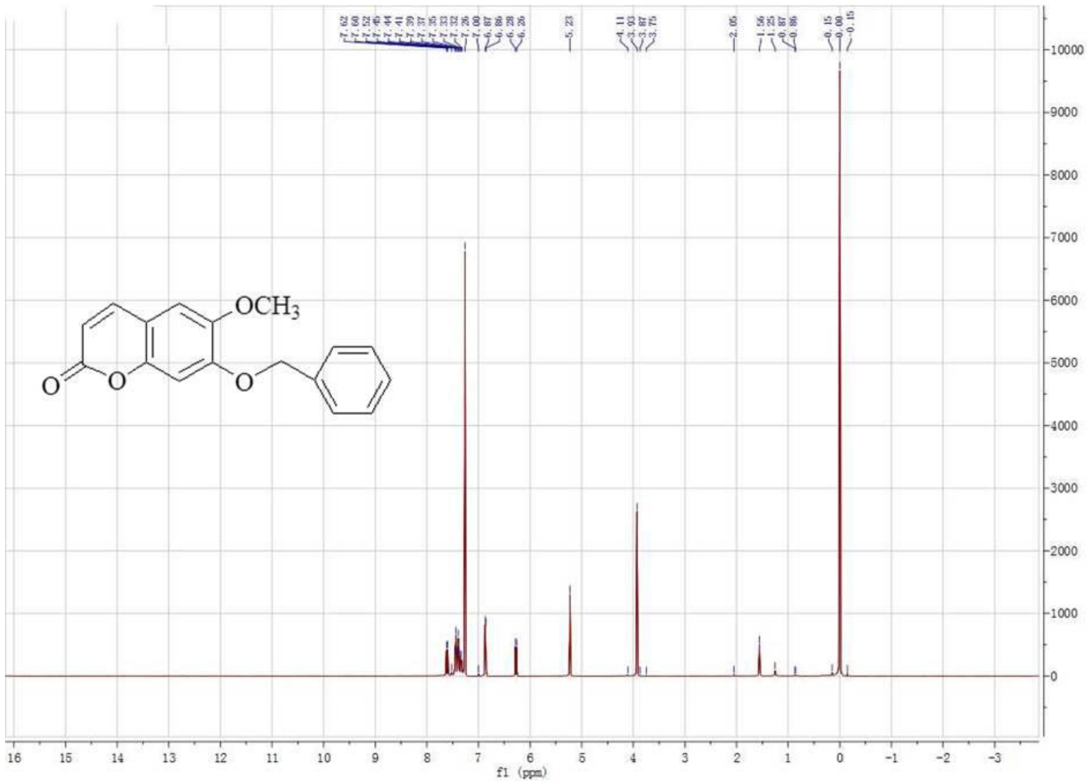

Figure S9. ^{1}H-NMR of 7-(benzyloxy)-6-methoxy-2*H*-chromen-2-one (**18**)

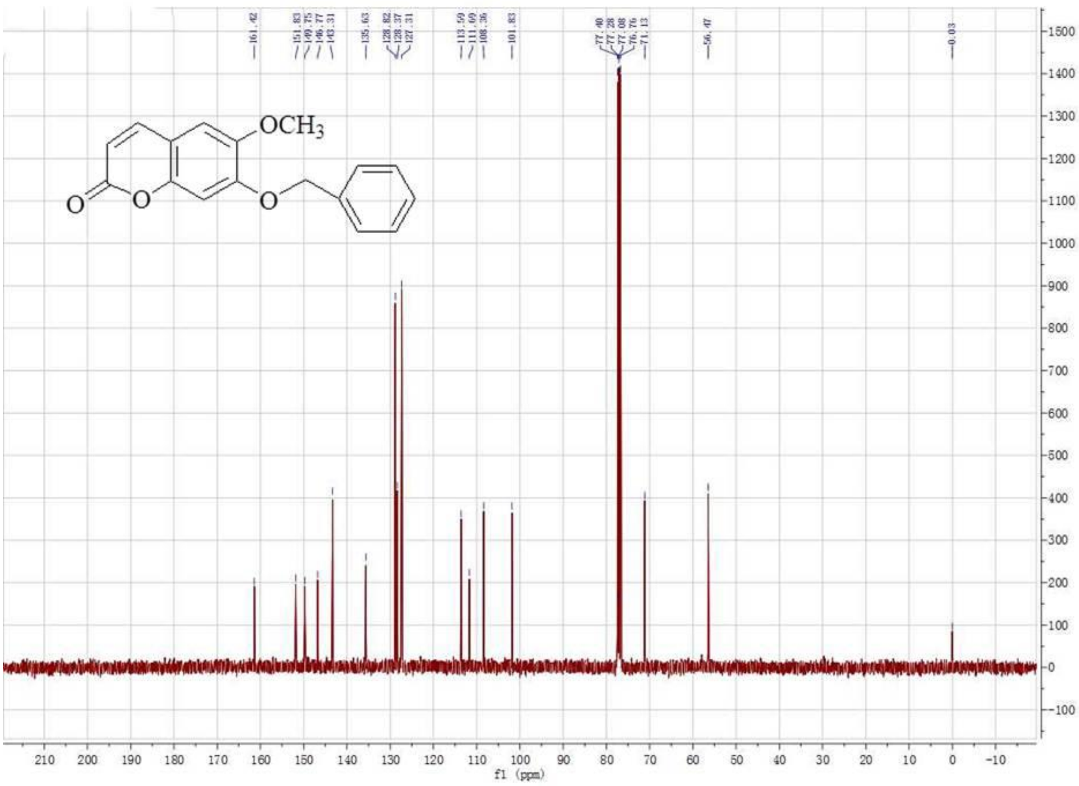

Figure S10. ^{13}C-NMR of 7-(benzyloxy)-6-methoxy-2*H*-chromen-2-one (**18**)

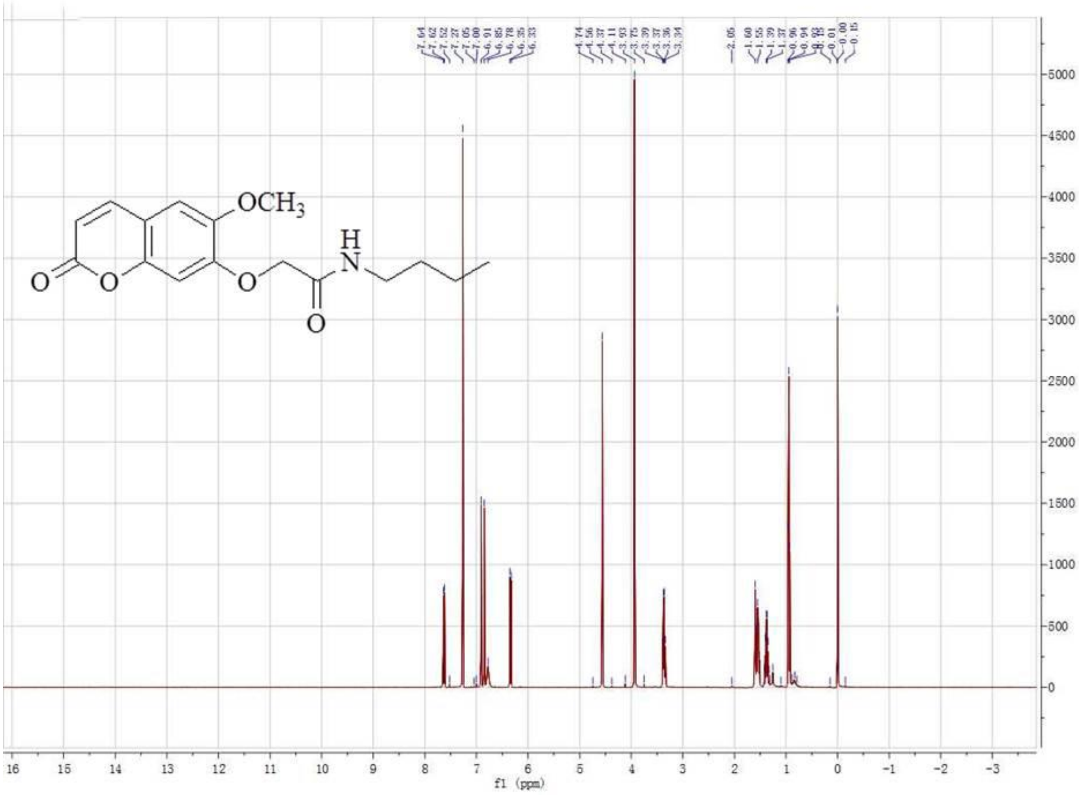

Figure S11. ^{1}H-NMR of *N*-butyl-2-(6-methoxy-2-oxo-2*H*-chromen-7-yloxy)acetamide (**30**)

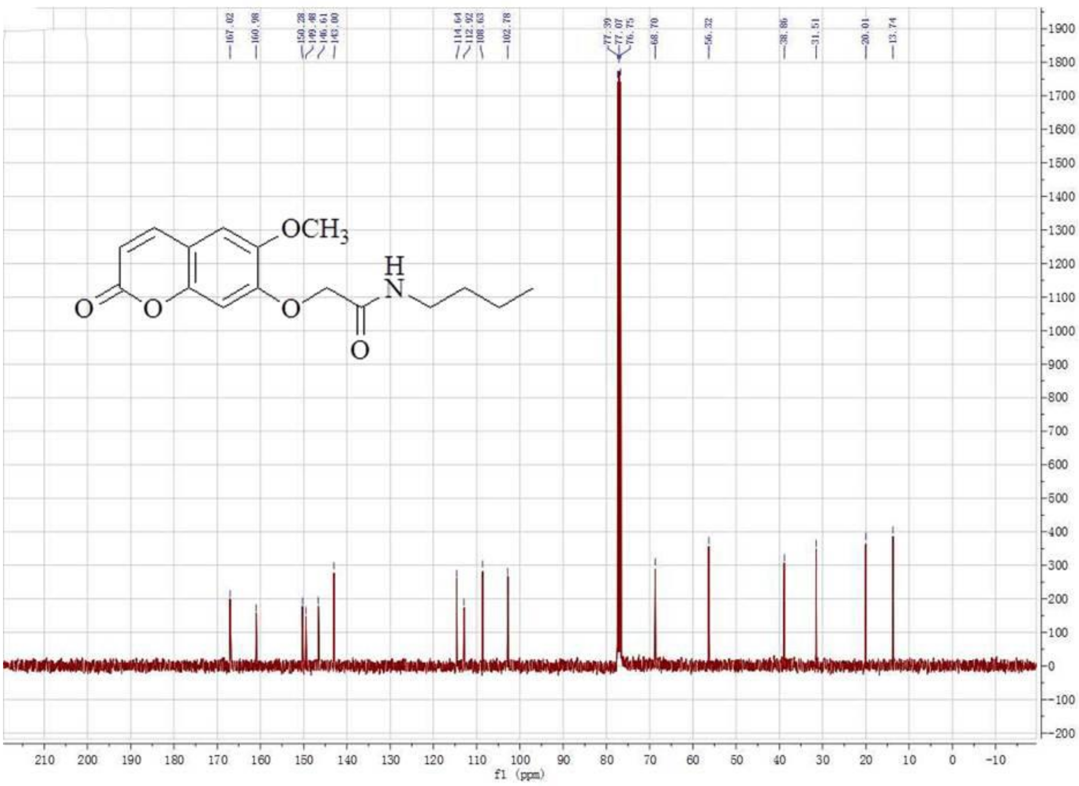

Figure S12. ^{13}C-NMR of *N*-butyl-2-(6-methoxy-2-oxo-2*H*-chromen-7-yloxy)acetamide (**30**)
